# Simultaneous analyses of urinary eicosanoids and related mediators identified tetranor-prostaglandin E metabolite as a novel biomarker of diabetic nephropathy

**DOI:** 10.1016/j.jlr.2021.100120

**Published:** 2021-09-22

**Authors:** Yoshifumi Morita, Makoto Kurano, Eri Sakai, Motoji Sawabe, Junken Aoki, Yutaka Yatomi

**Affiliations:** 1 Department of Clinical Laboratory, University of Tokyo Hospital, Tokyo, Japan; 2 Department of Molecular Pathology, Graduate School of Medical and Dental Sciences, Tokyo Medical and Dental University, Tokyo, Japan; 3 Department of Clinical Laboratory Medicine, Graduate School of Medicine, The University of Tokyo, Tokyo, Japan; 4 Department of Health Chemistry, Graduate School of Pharmaceutical Sciences, The University of Tokyo, Tokyo, Japan

**Keywords:** LC-MS/MS, urine, diabetic nephropathy, Eicosanoids, tetranor-PGEM, arachidonic acid, solid-phase extraction, maresin-1, leukotriene B4-ethanolamide, clinical stages, AEA, arachidonoyl ethanolamide, AUC, area under the curve, COX, cyclooxygenase, CSF, cerebrospinal fluid, CV, coefficient of variation, DN, diabetic nephropathy, GFR, glomerular filtration rate, LT, leukotriene, MRM, multiple reactions monitoring, OPLS, orthogonal projection to latent structure, PG, prostaglandin, ROC, receiver operating characteristic, SPE, solid-phase extraction, tetranor-PGEM, tetranor-prostaglandin E metabolite

## Abstract

Diabetic nephropathy is a major complication of diabetes mellitus, and thus novel biomarkers are desired to evaluate the presence and progression of diabetic nephropathy. In this study, we sought to identify possible metabolites related to diabetic nephropathy among urinary eicosanoids and related mediators. Using liquid chromatogram-tandem mass spectrometry, we optimized the lipid extraction from urine using the Monospin C18 as a solid-phase extraction cartridge and measured the urinary lipid mediators in 111 subjects with type 2 diabetes mellitus as well as 33 healthy subjects. We observed that 14 metabolites differed significantly among the clinical stages of nephropathy. Among them, levels of tetranor-prostaglandin E metabolite (tetranor-PGEM), an arachidonic acid metabolite, were significantly higher in subjects with stage 1 nephropathy than in healthy subjects and increased with the progression of nephropathy. We also observed that levels of maresin-1, a docosahexaenoic acid metabolite, and leukotriene B4-ethanolamide, an arachidonoyl ethanolamide metabolite, were significantly lower in subjects with stage 3–4 nephropathy than in healthy subjects and those with stage 1–2 nephropathy. Finally, using a comprehensive analysis of urinary eicosanoids and related mediators, we concluded that tetranor-PGEM was capable of discriminating clinical stages of nephropathy and thus useful as a novel biomarker for diabetic nephropathy.

Diabetic nephropathy (DN) is a major complication of diabetes (1) and effective treatment can reverse or delay the early progression of DN; thus, the early diagnosis of DN is important in the medical treatment of diabetes. The evaluation of albuminuria and the glomerular filtration rate (GFR) has been recommended by the Kidney Disease Improving Global Outcomes CKD Work Group (KDIGO) (2). However, several studies have reported that a rapid GFR decline can be seen in patients with normoalbuminuria (1, 3, 4, 5). Therefore, a novel biomarker for the diagnosis and staging of DN is needed.

Eicosanoids, such as prostaglandins (PGs) and leukotrienes (LTs), are widely known as potent bioactive lipids derived from the metabolism of arachidonic acid (AA) by cyclooxygenases (COX), lipoxygenases, cytochrome P450s, or nonenzymatic pathways (6). Eicosanoids are found in biological fluids and tissues including plasma (7, 8), cerebrospinal fluid (CSF) (9), and urine (10, 11), and many studies have reported their contributions to inflammation, autoimmunity, allergy, and cancer (11, 12). In addition to eicosanoids, lipid derivatives from ω-3 fatty acids, such as resolvins, have also been demonstrated to possess potent physiological properties (13). Metabolic derivatives are one type of candidate biomarker in diabetic kidney disease (14), and considering the relation between eicosanoids and their related mediators and inflammation, they could be useful biomarkers for DN.

To introduce the measurement of lipid mediators in blood samples, including eicosanoids and lysophospholipids, into clinical laboratory testing (15), artificially increased levels during or after sampling are a major issue that must be overcome. Since urine samples contain far fewer blood cells than blood samples, urine specimens are expected to be appropriate for the analysis of changes in lipid mediator levels. Sample preparation methods should be optimized for the measurement of eicosanoids and related mediators. To reduce ion suppression or enhancement and to purify the compounds, establishing the optimal procedure for solid-phase extraction (SPE) according for each matrix is necessary. Furthermore, when urine samples are used to identify biomarkers, 24-h urine samples are desirable, while spot urine testing and normalization according to the urinary creatinine level are often used to adjust for differences in dilution (16). A comparison of measured values between 24-h urine sampling and spot urine measurements adjusted according to the creatinine level is thus required.

With this background in mind, we first aimed to confirm the stability of eicosanoids and related mediators in urine samples and to optimize the sampling procedure according to the urine matrix for LC-MS/MS analysis. Then, we analyzed these lipid metabolites in urine samples collected from subjects with type 2 diabetes and healthy controls.

## Materials and methods

### Participants and samples

Residual urinary specimens after clinical examination were obtained from 111 participants with type 2 diabetes. Subjects who were renal transplant recipients or undergoing hemodialysis treatment were excluded. As a control group, 33 healthy participants without kidney disease and with urinary microalbumin (μAlb) levels below 30 mg/gCr and eGFR above 60 ml/min per 1.73 m^2^ were enrolled. We also enrolled 11 subjects with renal sclerosis who had antihypertension drugs and did not suffer from diabetes. The characteristics of the participants are shown in supplemental Table S1. We categorized the subjects with diabetes based on the stage of DN as determined using the 2014 classification for diabetic nephropathy published by the Research Group of Diabetic Nephropathy in Japan (2). Urine specimens were centrifuged at 1,700 *g* for 5 min, and the supernatants were stored at −80°C until measurement.

The current study was performed under the ethics guidelines laid down in the Declaration of Helsinki. Informed consent was obtained in writing from the diabetic subjects and in the form of an opt-out on our institution’s website from the control subjects. Participants who declined to be enrolled were excluded. This study was approved by the Institutional Research Ethics Committee of the Faculty of Medicine, The University of Tokyo (11158 and 2602).

### LC-MS/MS analysis

A comprehensive analysis method for eicosanoids has been described previously (17). The method utilizes an LC-8060 device, consisting of a quantum triple-quadrupole mass spectrometer (Shimadzu, Kyoto, Japan) and a software method package for the simultaneous analysis of 196 products with 18 deuterium internal standards (supplemental Table S2): tetranor-PGEM-d6, 6-keto-PGF1α-d4, TXB2-d4, PGF2α-d4, PGE2-d4, PGD2-d4, PGA2-d4, LTB4-d4, 14,15-DiHET-d11, 15-HETE-d8, 12-HETE-d8, 5-HETE-d8, 11,12-EET-d11, LTC4-d5, LTD4-d5, PAF-d4, OEA-d4, and AA-d8 (Cayman Chemical, Ann Arbor, MI). The eicosanoids were identified by multiple reactions monitoring (MRM) and were analyzed using LabSolutions software (Shimadzu).

### Sample preparation for LC-MS/MS analysis

Lipid extraction was performed using urinary specimens, and the method was validated using three SPE cartridges: Strata-X (10 mg/1 ml; Phenomenex, Torrance, CA), Oasis PRiME HLB (30 mg/1 ml; Waters Corporation, Milford, MA), and Monospin C18 (GL Sciences, Tokyo, Japan). Briefly, 40 μl of urine was mixed with 40 μl of methanol acidified with 0.1% formic acid and 10 ng/ml (final concentration) of the internal standards solutions; other than 2 ng/ml of OEA-d4 and 100 ng/ml of AA-d8 were added. The samples were sonicated for 3 min and centrifuged at 14,400 *g* for 10 min at 4°C. The supernatants were loaded onto each cartridge for SPE, and the analyte was eluted with 200 μl of methanol acidified with 0.1% formic acid.

To validate the SPE procedures, the repeatability and recovery rates were evaluated using three pooled urine samples with the addition of the following standard solutions: 10 ng/ml (final concentration) of tetranor-PGEM, 6-keto-PGF1α, TXB2, PGF2α, 8-iso-PGF2α, PGE2, PGD2, 15-keto-PGE2, PGF2α, 11-dehydro-TXB2, PGA2, LTB4, LTD4, 15-HETE, 12-HETE, 5-HETE, 11,12-EET, and PAF, and 100 ng/ml of AA.

### Diurnal fluctuation of urinary excretion of lipid mediators

To investigate the diurnal fluctuation of the urinary excretion of lipid metabolites, spot urine and 24-h urine specimens were collected from healthy volunteers (n = 5), and the measured values and the values of the ratio to urinary creatinine were compared with the values calculated from 24-h urine samples.

### Influences of incubation

To determine the influences of incubation on the concentrations of lipid mediators, three urine samples (normal urine, pyuria, and hematuria) were immediately centrifuged after sampling as we performed urinary chemical testing and divided into five tubes, and the samples were incubated at room temperature for 1, 3, or 24 h.

### Statistical analysis

The data were analyzed using SPSS (Chicago, IL) or SIMCA (MKS Umetrics). To compare the changes in the values according to incubation period, we performed a two-way ANOVA followed by the Tukey multiple comparison test. To compare the values according to the DN stage, we used the Kruskal–Wallis test followed by the Steel–Dwass test as a post-hoc test. *P* values less than 0.05 were deemed statistically significant for all the analyses. The orthogonal projection to latent structures (OPLS) was statistically analyzed using SIMCA to explore variables capable of predicting the DN stage.

## Results

### Precision of lipid extraction procedures for LC-MS/MS analysis

First, we optimized the SPE procedure for measuring lipid mediators in urinary specimens using three types of products. We added standard solutions of 19 metabolites to the urine samples and investigated the recovery rates (Table 1). Most of the analytes in the urine specimens containing the standard solutions were obtained with a high precision and a coefficient of variation (CV) of 2.4%–17.6% for Strata-X, 0.9%–60.3% for Oasis PRiME HLB, and 0.6%–9.9% for Monospin C18 (supplemental Table S3). The recovery rates (mean% [range%]) for the standard solutions were 102.8% (75.9%–127.7%) for Strata-X, 96.9% (11.1%–150.0%) for Oasis PRiME HLB, and 100.1% (89.7%–112.3%) for Monospin C18 (Table 1). Considering these results, we selected Monospin C18 as the optimal SPE cartridge for performing lipid extraction from urine samples.

**Table 1:** Recovery rates

Description	Strata-X	Oasis PRiME HLB	Monospin C18
tetranor-PGEM	75.9 ± 38.5	76.3 ± 43.7	106.0 ± 7.9
tetranor-PGDM	84.7 ± 47.5	76.3 ± 20.7	94.6 ± 28.4
6-keto-PGF1α	93.8 ± 4.5	91.5 ± 5.7	89.7 ± 8.0
TXB2	94.9 ± 0.7	90.0 ± 2.2	95.6 ± 4.1
8-iso-PGF2α	95.1 ± 3.0	98.8 ± 4.1	90.5 ± 2.5
PGE2	95.0 ± 2.0	98.3 ± 3.0	94.4 ± 4.2
PGD2	100.9 ± 3.9	98.1 ± 9.4	101.2 ± 3.4
15-keto-PGE2	111.0 ± 9.4	102.5 ± 7.3	105.3 ± 2.4
PGF2α	95.8 ± 3.6	101.5 ± 5.3	93.6 ± 2.7
11-dehydro-TXB2	105.7 ± 4.4	100.1 ± 1.0	99.3 ± 7.5
PGA2	111.6 ± 4.6	100.3 ± 3.3	98.5 ± 4.6
LTB4	104.5 ± 5.2	150.0 ± 11.6	109.1 ± 5.3
LTD4	113.7 ± 2.3	101.6 ± 6.9	104.5 ± 6.8
15-HETE	124.3 ± 15.3	120.8 ± 28.4	112.3 ± 20.0
12-HETE	96.3 ± 9.3	101.8 ± 8.0	110.5 ± 2.8
5-HETE	113.7 ± 12.4	117.8 ± 26.6	110.5 ± 8.7
11,12-EET	127.7 ± 11.6	105.4 ± 12.7	95.6 ± 10.5
PAF	106.3 ± 4.7	11.1 ± 9.6	93.2 ± 10.4
AA	101.8 ± 6.6	98.8 ± 3.0	97.0 ± 7.2

Data are shown as mean ± SD (n = 3).

### Effects of incubation on urinary levels of eicosanoids and related mediators

Next, we investigated the influences of incubation on the levels of urinary eicosanoids and related mediators using three urine samples (normal, pyuria, and hematuria). Twelve metabolites were detected in these samples: AA and its metabolites tetranor-PGEM, tetranor-PGDM, 20-hydroxy-PGE2, 15-keto-PGE2, 13,14-dihydro-15-keto-tetranor-PGD2, and 2,3-dinor-TXB2; lyso-PAF, a metabolite of PAF; PGF1α, a dihomo-γ-linolenic acid (DGLA) metabolite; LTB4-EA, an arachidonoyl ethanolamide (AEA) metabolite; and DHA and its metabolite maresin-1. Among them, 20-hydroxy-PGE2, lyso-PAF, DHA, and AA increased significantly in the pyuria sample and 20-hydroxy-PGE2 increased in the hematuria sample after several hours; these levels did not increase in the normal urine sample (supplemental Fig. S1). Regarding the other metabolites, although tetranor-PGEM, 15-keto-PGE2, 13,14-dihydro-15-keto-tetranor-PGD2, and LTB4-EA had decreased at 24-h and 2,3-dinor-TXB2 had increased at 3 h, the other metabolites remained unchanged for at least several hours, during which time period urine samples can usually be examined at room temperature during routine laboratory practices (supplemental Fig. S1).

### Diurnal fluctuations of the urinary excretion of lipid metabolites

We also investigated diurnal fluctuations in the urinary excretion of eicosanoids and related mediators using five samples obtained from healthy volunteers. Fifteen metabolites were detected in these samples: tetranor-PGEM, tetranor-PGDM, tetranor-PGAM, 13,14-dihydro-15-keto-PGF1α, 13,14-dihydro-15-keto-tetranor-PGD2, 13,14-dihydro-15-keto-tetranor-PGE2, PGF2α, and 15-keto-PGE2, all of which are AA metabolites; PGF1α; LTB4-EA; 9,10-DiHOME, an LA metabolite; OEA, an EA metabolite; lyso-PAF; DHA and maresin-1. The urinary levels of these metabolites were compared after normalization according to the urinary creatinine level and without normalization. The results for the diurnal fluctuations of urinary lipid metabolites are shown in supplemental Fig. S2, and the ratio of the levels in spot urine tests to those in 24-h urine samples and the ratio of the creatinine-normalized values for the spot urine tests to the levels for the 24-h urine samples are shown in supplemental Table S4. For metabolites other than lyso-PAF, OEA, and DHA, adjusting the values to the urinary creatinine level resulted in a smaller CV and was considered to be less susceptible to diurnal fluctuations at each sampling time, while the fluctuations varied greatly depending on individuals and metabolites (supplemental Fig. S2 and supplemental Table S4).

### Modulation of urinary eicosanoids and related mediators in subjects with diabetic nephropathy

So far, we optimized the lipid extraction method and confirmed the validity of creatinine adjustments. Using these methods, we next aimed to identify urinary eicosanoids and related mediators with the potential to be useful as biomarkers for the diagnosis and staging of DN. In clinical urinary specimens, 125 of 196 metabolites were detected, and 14 of these metabolites differed significantly between non-diabetes subjects and subjects with different stages of nephropathy.

**Fig. 1:**
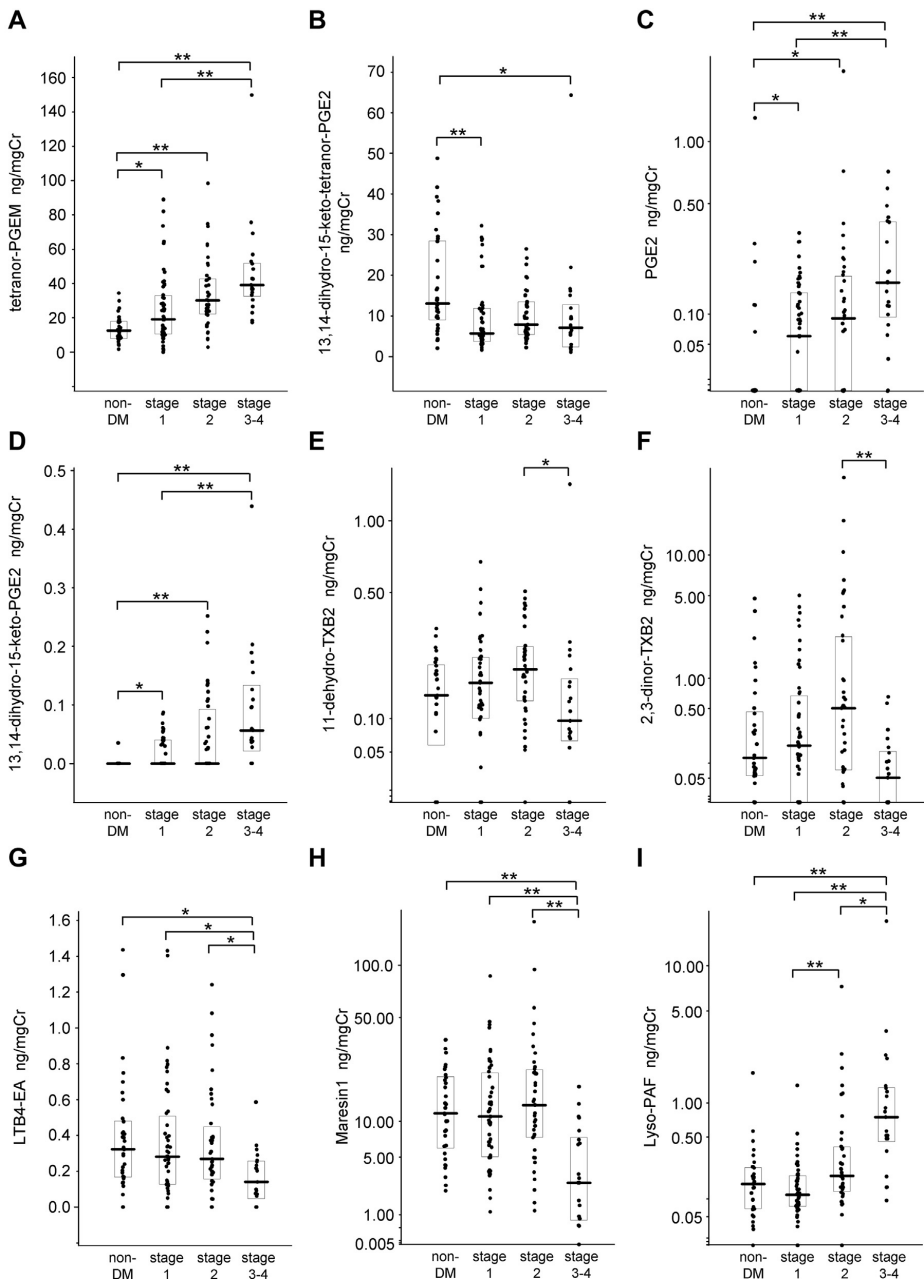
Modulation of urinary eicosanoids and related mediators according to the stages of diabetic nephropathy. We compared the urinary lipid metabolite levels between the subjects with diabetes and the control group. A: Tetranor-prostaglandin E metabolite (tetranor-PGEM); (B) 13,14-dihydro-15-keto-tetranor-PGE2; (C) PGE2; (D) 13,14-dihydro-15-keto-PGE2; (E) 11-dehydro-thromboxane B2 (11-dehydro-TXB2); (F) 2,3-dinor-TXB2; (G) leukotriene B4-ethanolamine (LTB4-EA); (H) maresin-1; (I) lyso-platelet activating factor (lyso-PAF). Data are shown as the median ± SE, ∗*P* < 0.05, ∗∗*P* < 0.01.

Regarding the AA derivatives, the levels of tetranor-PGEM, PGE2, and 13,14-dihydro-15-keto-PGE2 were significantly higher in subjects with stages 1, 2, and 3–4 of nephropathy than in the control group, and the levels were also significantly higher in the subjects with stage 3–4 nephropathy than in the subjects with stage 1 nephropathy (Fig. 1A, C, D); meanwhile, the level of 13,14-dihydro-15-keto-tetranor-PGE2 was significantly lower in the subjects with stage 1 and stage 3–4 nephropathy than in the control group (Fig. 1B). The level of 13,14-dihydro-15-keto-PGA2 was significantly higher in the subjects with stage 3–4 nephropathy than in both the control group and the subjects with stage 1 nephropathy (supplemental Fig. S3A). The level of PGD2 was also significantly higher in the subjects with stage 1 and stage 3–4 nephropathy than in the control group (supplemental Fig. S3B). Regarding the oxidized lipids derived from AA, the level of 8-iso-PGE2 was significantly higher only in the subjects with stage 1 nephropathy, compared with the control group (supplemental Fig. S3C), and the level of 5-iPF2α-VI was significantly lower in the subjects with stage 3–4 nephropathy than in the control group and the subjects with stage 1 and stage 2 nephropathy (supplemental Fig. S3D). Among the TXA2 derivatives, the levels of 11-dehydro-TXB2 and 2,3-dinor-TXB2 were significantly lower in the subjects with stage 3–4 nephropathy than in the subjects with stage 2 nephropathy (Fig. 1E, F).

In addition to the AA derivatives, we observed that several eicosanoid-related mediators were also modulated. The levels of LTB4-EA, a metabolite of AEA, and maresin-1, a DHA derivative, were significantly lower in the subjects with stage 3–4 nephropathy than in the control group and the subjects with stage 1 or 2 nephropathy (Fig. 1G, H). The level of 9,10-DiHOME, an LA derivative, was significantly lower in the subjects with stage 1 nephropathy than in the control group and the subjects with stage 2 nephropathy (supplemental Fig. S3E). The level of lyso-PAF, a metabolite of PAF, was significantly higher in the subjects with stage 3–4 nephropathy than in the other groups and was also significantly higher in the subjects with stage 2 nephropathy than in the subjects with stage 1 nephropathy (Fig. 1I).

### Association between urinary eicosanoids and their related mediators and clinical parameters in diabetic nephropathy

**Fig. 2:**
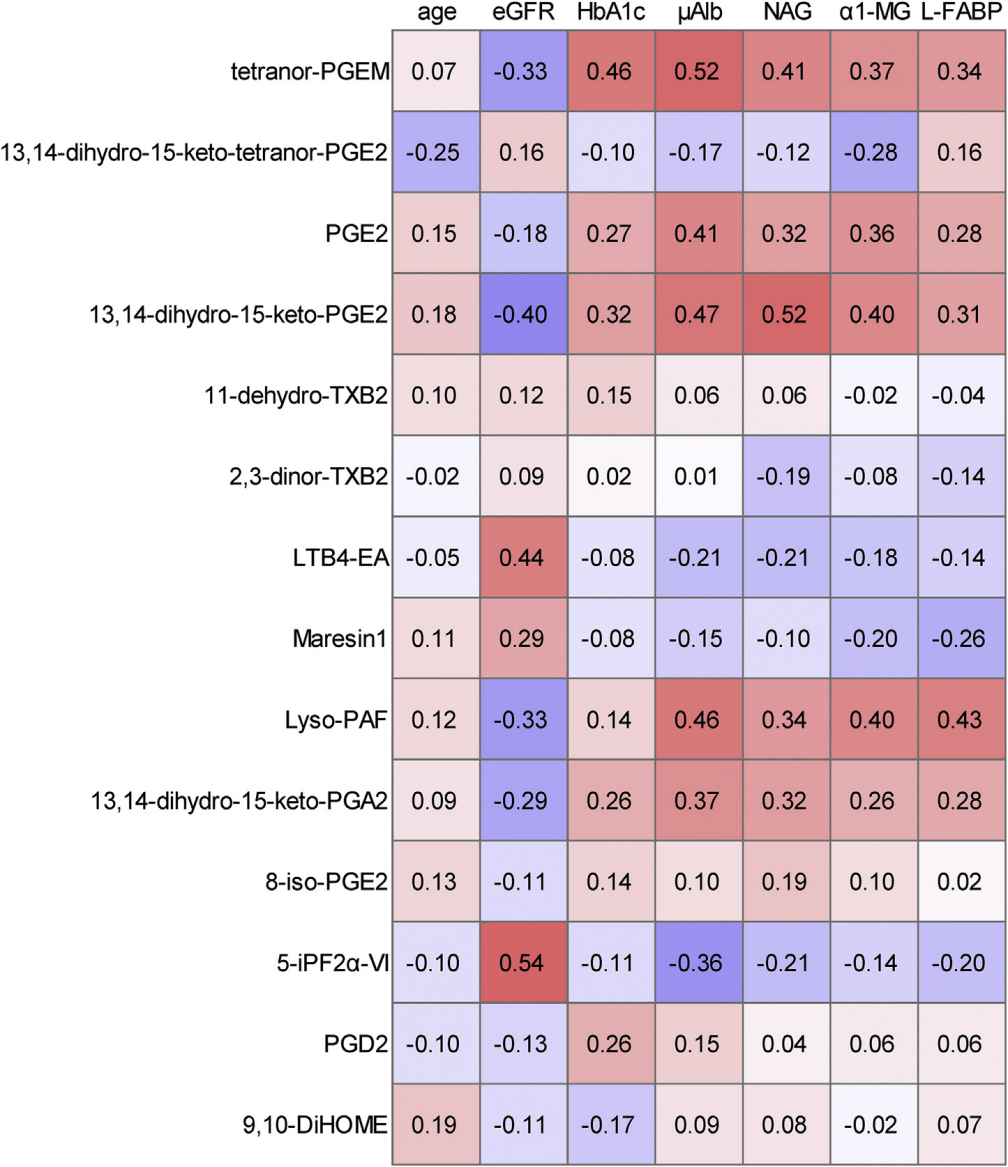
Association between urinary eicosanoids and related mediators and clinical parameters. Correlation analyses between urinary lipid metabolites and clinical parameters were performed. The correlation matrix is shown as Spearman’s rank correlation coefficient.

We investigated the association between clinical parameters and urinary metabolites that differed significantly according to DN stage, as listed in Fig. 1 and supplemental Fig. S3. Among the AA derivatives and their oxidized lipids, tetranor-PGEM, PGE2, and 13,14-dihydro-15-keto-PGA2 had the strongest positive correlations with μAlb, and 13,14-dihydro-15-keto-PGE2 had the strongest positive correlation with NAG (Fig. 2). In contrast, 13,14-dihydro-15-keto-tetranor-PGE2 was negatively correlated with α1-MG, and 5-iPF2α-VI was negatively correlated with μAlb, NAG, and L-FABP. The correlations between TXA2 derivatives and clinical biomarkers were not significant. Regarding the metabolites of AEA and DHA, LTB4-EA and maresin-1 had moderate positive correlations with eGFR (Fig. 2). LTB4-EA had negative correlations with μAlb, NAG, and α1-MG. Maresin-1 was negatively correlated with α1-MG and L-FABP.

### OPLS analysis of urinary metabolites in subjects with diabetic nephropathy

**Fig. 3:**
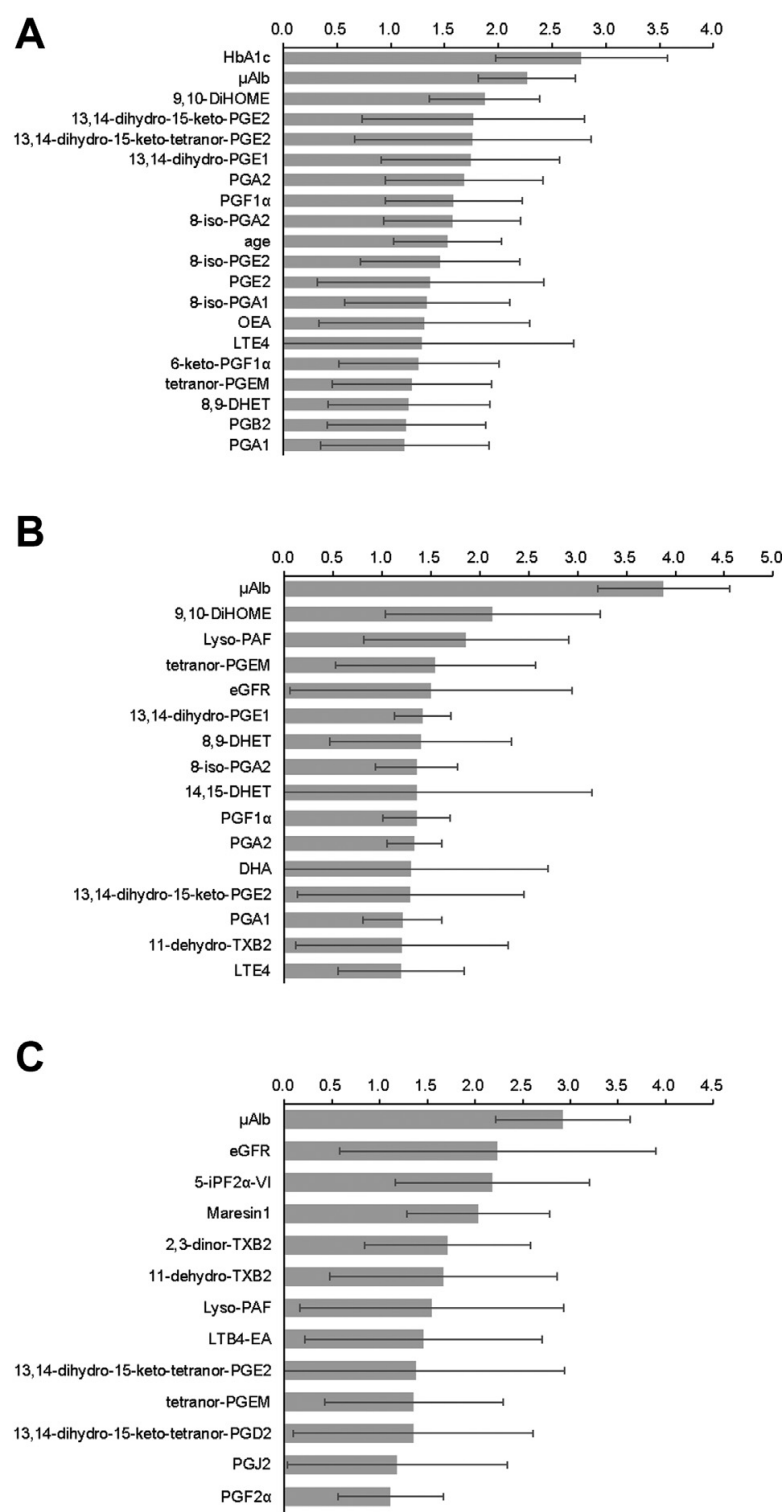
OPLS analysis for metabolites with variable importance in projection. The VIP scores of the OPLS-DA results are shown. A, Stage 1 nephropathy group versus control group, (B) stage 2 nephropathy group versus stage 1 nephropathy group, and (C) stage 3–4 nephropathy group versus stage 2 nephropathy group.

Next, we performed an OPLS analysis to identify markers capable of differentiating the presence or stage of DN. Using age, sex, eGFR, HbA1c, μAlb, and the metabolites detected in urinary samples as variables, the OPLS-DA model showed that 9,10-DiHOME, 13,14-dihydro-15-keto-PGE2, 13,14-dihydro-15-keto-tetranor-PGE2, 8-iso-PGE2, PGE2, and tetranor-PGEM were selected as significant contributing variables, following HbA1c and μAlb, for discriminating subjects with stage 1 nephropathy from the control group (Fig. 3A and supplemental Fig. S4). Regarding the variables capable of discriminating between subjects with stage 2 nephropathy from those with stage 1 nephropathy, 9,10-DiHOME, lyso-PAF, tetranor-PGEM, 13,14-dihydro-15-keto-PGE2, and 11-dehydro-TXB2 were selected, following μAlb (Fig. 3B and supplemental Fig. S5). We found that 5-iPF2α-VI, maresin-1, 2,3-dinor-TXB2, 11-dehydro-TXB2, lyso-PAF, and LTB4-EA were selected as significant variables following μAlb, allowing the discrimination of subjects with stage 3–4 nephropathy from those with stage 2 nephropathy (Fig. 3C and supplemental Fig. S6).

### ROC analysis of candidate metabolites contributing to nephropathy staging

We validated the diagnostic ability of the candidate metabolites selected by multigroup comparison and OPLS analysis using a receiver operating characteristic (ROC) curve analysis. The areas under the curve (AUCs) (95% confidence interval [CI]) are shown in Table 2. Tetranor-PGEM had the largest AUC of 0.791 (0.717–0.866) for discriminating subjects with stage 1 nephropathy from the control group (Model 1 in Table 2) and an AUC of 0.657 (0.561–0.753) for discriminating subjects with stage 2 from those with stage 1 (Model 2 in Table 2). Moreover, lyso-PAF and maresin-1 had high AUCs of 0.849 (0.761–0.938) and 0.823 (0.727–0.919), respectively, for discriminating subjects with stage 3–4 nephropathy from those with stage 2 nephropathy (Model 3 in Table 2). When the cutoff value was defined as 20.7 ng/mgCr based on Youden index to discriminate the stage 1 nephropathy or higher group from non-DM group, the sensitivity was 66.7% and the specificity was 87.9%. Similarly, the sensitivity and specificity were 83.3% and 67.9%, respectively, when the cutoff value of tetranor-PGEM was defined as 21.5 ng/mgCr to discriminate the stage 2 nephropathy or higher group, and 76.2% and 71.5%, respectively, when the cutoff value of tetranor-PGEM was defined as 30.4 ng/mgCr to discriminate the stage 3 nephropathy or higher group.

**Table 2:** ROC analysis

Model 1: Stage 1 Versus Non-DM				Model 2: Stage 2 Versus Stage 1				Model 3: Stage 3–4 Versus Stage 2			
Factor	Auroc	95% CI	*P* Value	Factor	Auroc	95% CI	*P* Value	Factor	Auroc	95% CI	*P* Value
μAlb	0.932	0.889–0.975	<0.001	μAlb	0.808	0.733–0.883	<0.001	μAlb	0.987	0.962–1.000	<0.001
tetranor-PGEM	0.791	0.717–0.866	<0.001	tetranor-PGEM	0.657	0.561–0.753	0.004	Lyso-PAF	0.849	0.761–0.938	<0.001
13,14-dihydro-15-keto-PGE2	0.733	0.652–0.814	<0.001	9,10-DiHOME	0.627	0.530–0.724	0.019	Maresin-1	0.823	0.727–0.919	<0.001
PGE2	0.721	0.623–0.819	<0.001	Lyso-PAF	0.602	0.504–0.701	0.059	5-iPF2α-VI	0.780	0.665–0.895	<0.001
13,14-dihydro-15-keto-tetranor-PGE2	0.714	0.615–0.813	<0.001					LTB4-EA	0.716	0.611–0.821	0.002
PGD2	0.651	0.537–0.766	0.008					2,3-dinor-TXB2	0.713	0.607–0.819	0.002
9,10-DiHOME	0.629	0.524–0.733	0.025					11-dehydro-TXB2	0.642	0.511–0.773	0.038
8-iso-PGE2	0.622	0.525–0.719	<0.05								

### Urinary tetranor-PGEM levels might contribute to discriminate diabetic nephropathy from renal sclerosis and predict the prognosis of diabetic nephropathy

Finally, we investigated the possible usefulness of urinary tetranor-PGEM levels as a urinary biomarker. When we measured 11 subjects with renal sclerosis, which is sometimes difficult to be clinically discriminated from DN. All of the subjects with renal sclerosis had antihypertension drugs and did not suffer from diabetes. While the proteinuria levels and eGFR of the sclerosis group were similar to DN stage 3–4 group (supplemental Fig. S7A, B), tetranor-PGEM was significantly lower in the sclerosis group than stage 3–4 nephropathy group (supplemental Fig. S7C).

We also investigated whether tetranor-PGEM would contribute to predict prognosis. We measured the levels of tetranor-PGEM in the urine samples, which had been collected 2 years ago from 39 diabetic subjects enrolled in the present study. Although there was no significant association between the progression of DN stage for 2 years and the tetranor-PGEM levels in the urine collected 2 years ago, the decline of eGFR level for the last 2 years was significantly correlated with the tetranor-PGEM levels in urine collected 2 years ago (supplemental Fig. S8).

## Discussion

The establishment of a reliable biomarker for the diagnosis and prognosis of DN is necessary, and we attempted to perform a comprehensive analysis using LC-MS/MS focusing on urinary eicosanoids and related mediators.

First, we optimized the lipid extraction procedures for urinary specimens. Validation of the SPE procedures for lipid extraction showed that the use of the Monospin C18 resulted in greater precision and higher recovery rates (Table 1 and supplemental Table S3). SPE procedures are commonly performed for lipid extraction, but the recovery rates can differ depending on the filler in the cartridges. Additionally, the levels of most of the compounds that were detected in the urinary specimens did not change with incubation at room temperature, although the levels of 20-hydroxy-PGE2, lyso-PAF, DHA, and AA tended to increase over time (supplemental Fig. S1). Since the levels of these lipids increased when left at room temperature in the centrifugated supernatants, prompt measurement or freezing samples will be required, when precise measurement of their concentrations is necessary. The present study is the first to evaluate the effects of incubation on the measurement of eicosanoids and related metabolites in urine samples.

Next, we analyzed the modulation of eicosanoids and related mediators in subjects with DN and investigated whether these lipid mediators could be used as biomarkers for the diagnosis and staging of DN. A schematic figure of the PGE metabolite pathway is shown in the supplemental materials (supplemental Fig. S9). Regarding AA and its related metabolites, we found that the level of tetranor-PGEM was significantly higher in subjects with stage 1 nephropathy than in the control group and increased with the progression of DN (Fig. 1A). Tetranor-PGEM is the major metabolite of PGE1 and PGE2, and an association between tetranor-PGEM and obesity-associated insulin resistance has been reported (18). The present study is the first to show the modulation of urinary tetranor-PGEM levels in subjects with DN. Moreover, the levels of PGE2 and 13,14-dihydro-15-keto-PGE2, which are located upstream in the metabolic pathway of tetranor-PGEM, also increased with the progression of DN (Fig. 1C, D). Concordantly, basic studies have shown that PGE2 and the EP4 receptor pathway, which is activated by PGE2, induce the progression of tubule interstitial fibrosis and albuminuria (19). The present study also showed that the urinary levels of PGE2 and its metabolites were correlated with urinary biomarkers such as μAlb and NAG (Fig. 2), suggesting that PGE2 and its metabolites might be associated with the enhancement of inflammation and RTE apoptosis in the kidneys. Although the reason why the modulation of 13,14-dihydro-15-keto-tetranor-PGE2 differs from that of other metabolites of PGE2 remains unclear, the metabolic process from 13,14-dihydro-15-keto-tetranor-PGE2 to tetranor-PGE2 might be accelerated in subjects with DN. The level of 5-iPF2α-VI, which is a regioisomeric isoprostane formed from AA and is a reliable biomarker of oxidative stress, also decreased during the progression of DN, which at first glance seems contrary to the hypothesis that oxidation might be involved in the pathogenesis of DN. Urinary L-FABP is known to be a biomarker of oxidative stress, and its levels increase with the progression of DN; meanwhile, 5-iPF2α-VI was negatively correlated with L-FABP. We speculated that the modulation of 5-iPF2α-VI in urine might differ from that in blood. Among the components of TXA2 metabolism, the levels of 11-dehydro-TXB2 and 2,3-dinor-TXB2 were lower in the stage 3–4 nephropathy group than in the stage 2 group, the decline in renal function might result in the impaired excretion of TXA2 metabolites. Regardless, our findings suggest that the prostaglandin biosynthesis pathways may be activated during the progression of nephropathy.

Regarding the other metabolites, LTB4-EA (an AEA metabolite), maresin-1 (a DHA metabolite), 9,10-DiHOME (an LA metabolite), and lyso-PAF (a PAF metabolite) were modulated among both the nondiabetes subjects and the subjects with DN. Both LTB4-EA and maresin-1 play a role in anti-inflammation, and our results showed that the levels of both of these metabolites decreased with the progression of DN. LTB4 is known as an inflammatory factor that is mediated through BLT1 and BLT2, and LTB4-EA is a potent antagonist of BLT1 (20). However, since the urinary levels of LTB4 and its related metabolites, such as 18-carboxy-LTB4 and 20-hydroxy-LTB4, did not differ significantly with the DN stage, the role of urinary LTB4-EA remains controversial. Maresin-1 is known as a proresolution lipid mediator derived from DHA and is reportedly associated with lung injury, pancreatitis, and arthritis (21, 22, 23); however, an association between maresin-1 and DN has not been previously reported. Proresolving lipid mediators such as resolvins are reportedly important for the management of diabetes and its complications (24), while resolvin D1, D2, D3, D4, and D5, which were monitored using comprehensive methods in the present study, were almost undetectable. In summary, the levels of LTB4-EA and maresin-1 tended to decrease with the progression of DN, suggesting that inflammation might result in a decrease in anti-inflammatory substances. Lyso-PAF is derived from PAF and its synthesis is reportedly accelerated in polymorphonuclear leukocytes from streptozotocin-induced diabetic rats (25). We found that the urinary lyso-PAF levels increased with the progression of DN and were positively correlated with urinary markers, suggesting that urinary lyso-PAF might be an indicator of inflammation in the kidneys of human diabetic subjects.

Finally, to establish possible biomarkers for the diagnosis and staging of DN, we performed OPLS and ROC analyses and found that tetranor-PGEM might be a novel candidate marker for discriminating between healthy subjects and subjects with stage 1 nephropathy as well as between stage 2 and stage 1 nephropathy. For the clinical application in the future, it is necessary to determine the threshold value of tetranor-PGEM to distinguish among stages of nephropathy. At present, we should admit that the ability of tetranor-PGEM level to discriminate the stage of diabetic nephropathy was inferior to the present clinical marker such as urinary albumin and protein levels since the stages of diabetic nephropathy are determined on these markers. Moreover, the issue of the wide distribution of tetranor-PGEM should be resolved in the future, for example, by adjustment for some related lipid compounds. However, although the results are preliminary at present, the urinary levels of tetranor-PGEM might be useful to discriminate advanced diabetic nephropathy from renal sclerosis (supplemental Fig. S7) and to predict the prognosis of diabetic nephropathy (supplemental Fig. S8). Since several studies have attempted to prevent the progression of DN by inhibiting the PG synthesis pathway (26, 27), the measurement of urinary tetranor-PGEM might be useful as a noninvasive marker for estimating the effects of inhibitors of PG synthesis. Moreover, we found that lyso-PAF, maresin-1, and LTB4-EA might be candidate markers for the progression of nephropathy. Since DHA/EPA intake reportedly suppresses damage to distal tubular cells (28) and the correlation analyses in the present study showed that maresin-1 was not correlated with μAlb but was negatively correlated with α1-MG and L-FABP, maresin-1, which is derived from DHA, might reflect the degree of tubular injury.

The main limitation of the present study is that this was an observational study, and follow-up studies are needed to investigate whether changes in the levels of eicosanoids and related mediators reflect the prognosis of nephropathy. In addition, eicosanoids and related metabolites are known to be influenced by medications and the subjects in the present study had several comorbidities and various treatments. When we analyzed the subjects treated with RAS inhibitors or platelet inhibitors separately, the significant elevation of the tetranor-PGEM levels was observed only in those who had not taken antiplatelet inhibitors (supplemental Tables S5 and S6). Furthermore, if these lipid mediators play a role in inflammation or anti-inflammation in the kidney, it is essential to investigate whether their modulation is specific to DN.

In conclusion, the comprehensive analysis of urinary eicosanoids and related mediators may contribute to the search for reliable biomarkers for the diagnosis and prognosis of DN, and our findings suggest that tetranor-PGEM might be a potential biomarker for DN.

## Data availability

The datasets generated or analyzed in the current study will be made available upon reasonable request.

## Supplemental data

This article contains supplemental data.

## Conflict of interest

The authors declare that they have no conflicts of interest with the contents of this article.

## Supplemental data

Supplemental Figures S1–S9 and Tbales S1–S6
