# Supplementary material for: Simultaneous analyses of urinary eicosanoids and related mediators identified tetranor-prostaglandin E metabolite as a novel biomarker of diabetic nephropathy
Source: J Lipid Res. 2021 Sep 22;62:100120. doi: 10.1016/j.jlr.2021.100120 (PMC8515300; doi:10.1016/j.jlr.2021.100120)
Supplement: Supplemental Figures S1–S9 and Tbales S1–S6 [file mmc1.pdf]

Supplemental Tables

Table S1. Clinical characteristics of participants

		non-DM	Stage 1	Stage 2	Stage 3-4
n		33	51	39	21
Age, years		55.8±9.9	64.1±12.0*	67.3±11.3*	61.9±15.2*
Sex (M/F)		16/17	28/23	26/13	15/6
SBP, mmHg		N/A	130.2±12.8	134.7±10.9	135.9±16.4
DBP, mmHg		N/A	75.6±8.6	73.6±15.5	74.9±14.1
HbA1c, %		5.6±0.3	7.6±1.4*	7.7±1.1*	8.0±1.6*
eGFR		76.0±12.6	72.6±16.3	61.9±19.1*†	34.9±21.7*†‡
μAlb, mg/gCr (median [range])		4.0 [2.1, 21.4]	9.2 [3.7, 29.8] *	81.7 [30.6, 281.1] *†	859.1 [42.5, 9097.5] *†‡
NAG, U/gCr (median [range])		3.1 [1.2, 10.7]	5.1 [0.0, 25.1] *	8.3 [2.9, 49.1] *†	10.6 [4.9, 38.5] *†
α1-MG, mg/gCr (median [range])		1.6 [0.6, 10.5]	5.0 [0.4, 15.8] *	9.2 [1.6, 55.0] *†	35.4 [6.1, 157.2] *†‡
L-FABP, μg/gCr (median [range])		1.5 [0.2, 8.3]	1.5 [0.0, 6.7]	4.3 [0.0, 276.6] *†	43.8 [2.3, 359.4] *†‡
Urinary sediment					
Renal tubular cells (%)	(-)	33 (100.0)	48 (96.0)	35 (89.7)	16 (76.2)
	<1 /HPF	0 (0.0)	2 (4.0)	3 (7.7)	4 (19.0)
	1≤ /HPF	0 (0.0)	0 (0.0)	1 (2.6)	1 (4.8)
Oval fat body (%)	(-)	33 (100.0)	50 (100.0)	39 (100.0)	14 (66.7)
	1-9 /WF	0 (0.0)	0 (0.0)	0 (0.0)	4 (19.0)
	10≤ /WF	0 (0.0)	0 (0.0)	0 (0.0)	3 (14.3)
Hyaline casts (%)	<10 /WF	33 (100.0)	44 (88.0)	29 (74.4)	11 (52.4)
	10-49 /WF	0 (0.0)	4 (8.0)	4 (10.3)	6 (28.6)
	50≤ /WF	0 (0.0)	2 (4.0)	6 (15.4)	4 (19.0)

Epithelial casts (%)	<5 /WF	33 (100.0)	50 (100.0)	38 (97.4)	20 (95.2)
	5≤ /WF	0 (0.0)	0 (0.0)	1 (2.6)	1 (4.8)
Granular casts (%)	<5 /WF	33 (100.0)	50 (100.0)	37 (94.9)	16 (76.2)
	5-29 /WF	0 (0.0)	0 (0.0)	2 (5.1)	1 (4.8)
	30≤ /WF	0 (0.0)	0 (0.0)	0 (0.0)	4 (19.0)
Waxy casts (%)	(-)	33 (100.0)	50 (100.0)	39 (100.0)	16 (76.2)
	(+)	0 (0.0)	0 (0.0)	0 (0.0)	5 (23.8)
Fatty casts (%)	(-)	33 (100.0)	50 (100.0)	39 (100.0)	12 (57.1)
	(+)	0 (0.0)	0 (0.0)	0 (0.0)	9 (42.9)
Vacuolar denatured casts (%)	(-)	33 (100.0)	50 (100.0)	39 (100.0)	18 (85.7)
	(+)	0 (0.0)	0 (0.0)	0 (0.0)	3 (14.3)
Comorbidities, n (%)					
Cardiovascular disease		1 (3.0)	14 (27.5)	9 (23.1)	7 (33.3)
Cerebrovascular disease		0 (0.0)	4 (7.8)	5 (12.8)	1 (4.8)
Hypertension		0 (0.0)	20 (39.2)	22 (56.4)	13 (61.9)
Dyslipidemia		1 (3.0)	31 (60.8)	21 (53.8)	17 (81.0)
Drugs in past six months					
Diabetes drugs					
Insulin		0 (0.0)	13 (25.5)	11 (28.2)	8 (38.1)
GLP-1		0 (0.0)	2 (3.9)	1 (2.6)	4 (19.0)
Biguanide		0 (0.0)	9 (17.6)	10 (25.6)	3 (14.3)
Thiazolidine		0 (0.0)	3 (5.9)	5 (12.8)	0 (0.0)
Sulphonylureas		0 (0.0)	12 (23.5)	14 (35.9)	3 (14.3)
Glinides		0 (0.0)	4 (7.8)	3 (7.7)	1 (4.8)
DPP-4 inhibitors		0 (0.0)	26 (51.0)	25 (64.1)	13 (61.9)
α-GI		0 (0.0)	8 (15.7)	4 (10.3)	4 (19.0)
SGLT2 inhibitors		0 (0.0)	5 (9.8)	7 (17.9)	3 (14.3)

No diabetes drugs	0 (0.0)	7 (13.7)	3 (7.7)	1 (4.8)
Antihypertensive drugs	1 (3.0)	23 (45.1)	29 (74.4)	
ARB/ACE inhibitors	0 (0.0)	14 (27.5)	20 (51.3)	17 (81.0)
Calcium channel blocker	1 (3.0)	16 (31.4)	16 (41.0)	13 (61.9)
diuretics	0 (0.0)	3 (5.9)	9 (23.1)	6 (28.6)
β-blocker	0 (0.0)	3 (5.9)	5 (12.8)	5 (23.8)
Other antihypertensive	0 (0.0)	0 (0.0)	0 (0.0)	2 (9.5)
Lipid lowering drugs	1 (3.0)	32 (62.7)	20 (51.3)	16 (76.2)
Platelet inhibitors	1 (3.0)	7 (13.7)	12 (30.8)	9 (42.9)
Aspirin	1 (3.0)	4 (7.8)	9 (23.1)	7 (33.3)
Clopidogrel sulfate	0 (0.0)	1 (3.0)	1 (2.6)	0 (0.0)
Others	0 (0.0)	2 (3.9)	2 (5.1)	2 (9.5)

*: $P < 0.05$ vs. non-DM, †: $P < 0.05$ vs. stage 1, ‡: $P < 0.05$ vs. stage 2

Table S2. Lipid metabolites in a method package (Shimadzu, version 3)

No.	Category	Compounds	IS group
1	AA	tetranor-PGFM	1
2	IS	tetranor-PGEM-d6	1
3	AA	tetranor-PGEM	1
4	AA	tetranor-PGDM	1
5	AA	tetranor-PGJM	1
6	AA	tetranor-PGAM	1
7	AA	20-hydroxy-PGF2α or 19-hydroxy-PGF2α	2
8	AA	20-hydroxy-PGE2	2
9	AA	18-carboxy-dinor-LTB4	2
10	EPA	δ17-6-keto-PGF1α	2
11	AA	13,14-dihydro-15-keto-tetranor-PGF1β	2
12	DGLA	2,3-dinor-TXB1	3
13	AA	2,3-dinor-8-iso-PGF2α	2
14	AA	2,3-dinor-TXB2	3
15	AA	13,14-dihydro-15-keto-tetranor-PGF1α	2
16	EA	2,3-dinor-11β-PGF2α	2
17	IS	6-keto-PGF1α-d4	2
18	AA	6-keto-PGF1α	2
19	AA	13,14-dihydro-15-keto-tetranor-PGD2	2
20	EPA	Resolvin E1	2
21	AA	20-carboxy-LTB4	2
22	EA	PGF2α-EA	2
23	DGLA	6-keto-PGE1	2
24	EA	PGE2-EA	2
25	EPA	8-iso-PGF3α	2
26	EPA	TXB3	3
27	AA	20-hydroxy-LTB4	2
28	AA	PGE1-EA	2
29	AA	11-dehydro-2,3-dinor-TXB2	2
30	AA	13,14-dihydro-15-keto-tetranor-PGE2	2
31	DGLA	2,3-dinor-PGE1	2
32	EA	PGD2-EA	2
33	AA	6,15-diketo-13,14-dihydro-PGF1α	2
34	EPA	PGF3α	4
35	AA	iPF2α-IV	4
36	AA	8-iso-15(R)-PGF2α	4
37	DGLA	TXB1	3
38	AA	8-iso-PGF2α	4
39	IS	TXB2-d4	3

40	EPA	11-dehydro-TXB3	4
41	AA	TXB2	3
42	EPA	PGE3	4
43	DGLA	8-iso-PGF1α	4
44	AA	11β-PGF2α	4
45	AA	5-iPF2α-VI	4
46	EPA	PGD3	4
47	AA	8-iso-15-keto-PGF2α	4
48	IS	PGF2α-d4	4
49	AA	PGF2α	4
50	DGLA	PGF1α	4
51	AA	8-iso-13,14-dihydro-15-keto-PGF2α	4
52	EPA	LXA5	5
53	AA	8-iso-PGE2	5
54	IS	PGE2-d4	5
55	AA	PGE2	5
56	AA	11-dehydro-TXB2	5
57	DGLA	8-iso-PGE1	5
58	AA	15-keto-PGF2α	5
59	AA	11β-PGE2	5
60	DHA	Resolvin D3	5
61	AA	5S,14R-LXB4	6
62	AA	PGK2	6
63	IS	PGD2-d4	6
64	DGLA	PGE1	6
65	AA	PGD2	6
66	DGLA	PGD1	6
67	AA	15-keto-PGF1α	6
68	AA	11β-13,14-dihydro-15-keto-PGF2α	6
69	AA	15-keto-PGE2	6
70	DHA	Resolvin D2	6
71	AA	13,14-dihydro-PGF1α	6
72	DGLA	13,14-dihydro-PGE1	6
73	AA	14,15-LTC4	7
74	AA	13,14-dihydro-15-keto-PGF2α	6
75	AA	5S,6R-LXA4	6
76	AA	13,14-dihydro-15-keto-PGE2	6
77	DHA	Resolvin D1	6
78	AA	5S,6S-LXA4	6
79	AA	14,15-LTE4	7
80	ADA	1a,1b-dihomo-PGF2α	6

81	AA	13,14-dihydro-15-keto-PGD2	6
82	EA	LTB4-EA	6
83	IS	LTC4-d5	7
84	AA	LTC4	7
85	AA	11-trans-LTC4	7
86	IS	LTD4-d5	8
87	AA	LTD4	8
88	DHA	Resolvin D4	9
89	AA	LTE4	7
90	AA	LTF4	7
91	AA	8-iso-PGA2	9
92	DGLA	13,14-dihydro-15-keto-PGD1	9
93	AA	11-trans-LTD4	8
94	DGLA	8-iso-PGA1	9
95	IS	PGA2-d4	9
96	AA	PGA2	9
97	EPA	LTB5	9
98	AA	PGJ2	9
99	AA	11-trans-LTE4	7
100	AA	PGB2	9
101	DGLA	PGA1	9
102	AA	8,12-iso-iPF2α-VI-1,5-lactone	10
103	AA	8,15-DiHETE	10
104	EPA	17,18-DiHETE	10
105	AA	6-trans-LTB4	10
106	AA	5,15-DiHETE	10
107	AA	13,14-dihydro-15-keto-PGA2	10
108	DHA	Maresin1	10
109	IS	LTB4 d4	10
110	AA	LTB4	10
111	DHA	10,17-DiHDHA	10
112	AA	13,14-dihydro-15-keto PGJ2	10
113	DHA	Resolvin D5	10
114	EPA	14,15-DiHETE	11
115	DHA	7,17-hydroxy-DPA	11
116	LA	12,13-DiHOME	11
117	LA	9,10-DiHOME	11
118	AA	12-keto-LTB4	11
119	EPA	5,6-DiHETE	11
120	AA	tetranor-12-HETE	11
121	AA	N-acetyl-LTE4	11

122	IS	14,15-DiHET-d11	11
123	AA	LTB3	11
124	DHA	19,20-DiHDPA	11
125	AA	14,15-DHET	11
126	AA	12-HHT	11
127	AA	11,12-DHET	11
128	AA	8,9-DHET	11
129	AA	20-carboxy-AA	11
130	ALA	9-HOTrE	11
131	EA	14,15-EET-EA	11
132	AA	5,6-DHET	11
133	ALA	13-HOTrE	11
134	EPA	18-HEPE	11
135	AA	19-HETE	11
136	AA	15-deoxy-delta-12,14-PGJ2	11
137	AA	20-HETE	12
138	EA	11,12-EET-EA	12
139	EPA	15-HEPE	12
140	EPA	11-HEPE	12
141	AA	18-HETE	12
142	EPA	8-HEPE	12
143	ALA	13-HpOTrE	12
144	EA	8,9-EET-EA	12
145	EPA	9-HEPE	12
146	AA	17-HETE	12
147	EPA	12-HEPE	12
148	EA	5,6-EET-EA	12
149	AA	16-HETE	12
150	EPA	5-HEPE	12
151	other	Lyso-PAF	12
152	EPA	15-HpEPE	12
153	LA	13-HODE	12
154	LA	9-HODE	12
155	EPA	12-HpEPE	12
156	DHA	20-HDHA	12
157	IS	15-HETE-d8	12
158	EPA	5-HpEPE	12
159	AA	15-HETE	12
160	EPA	17,18-EpETE	12
161	LA	9-HpODE	12
162	LA	13-KODE	12

163	LA	13-HpODE	12
164	DHA	16-HDHA	13
165	DHA	17-HDHA	13
166	LA	9-KODE	13
167	AA	11-HETE	13
168	DHA	13-HDHA	13
169	DHA	10-HDHA	13
170	AA	8-HETE	13
171	IS	12-HETE-d8	13
172	DHA	14-HDHA	13
173	AA	15-KETE	13
174	AA	15-HpETE	13
175	EPA	14,15-EpETE	13
176	AA	12-HETE	14
177	DHA	11-HDHA	14
178	DHA	7-HDHA	14
179	AA	9-HETE	14
180	IS	5-HETE-d8	14
181	DHA	8-HDHA	14
182	AA	5-HETE	14
183	DHA	17-HpDHA	14
184	DGLA	15-HETrE	14
185	IS	PAF-d4	15
186	other	PAF	15
187	DGLA	8-HETrE	14
188	AA	12-HpETE	14
189	AA	12-KETE	14
190	AA	5,6-DHET-lactone	14
191	DHA	4-HDHA	14
192	AA	5-HpETE	14
193	LA	12,13-EpOME	14
194	LA	9,10-EpOME	14
195	DHA	19,20-EpDPA	14
196	AA	14,15-EET	16
197	AA	5-KETE	16
198	other	Azelaoyl-PAF	15
199	EDA	11-HEDE	16
200	DHA	16,17-EpDPA	16
201	EDA	15-HEDE	16
202	IS	11,12-EET-d11	16
203	EA	AEA	17

204	AA	11,12-EET	16
205	DGLA	5-HETrE	16
206	AA	8,9-EET	16
207	AA	5,6-EET	16
208	EDA	15-KEDE	17
209	IS	OEA-d4	17
210	EA	OEA	17
211	EPA	EPA	18
212	DHA	DHA	18
213	IS	AA-d8	18
214	AA	AA	18

AA, arachidonic acid; ALA, α-linolenic acid; EA, ethanolamide; EDA, eicosadienoic acid; EPA, eicosapentaenoic acid; DGLA, dihomo-γ-linolenic acid; DHA, docosahexaenoic acid; IS, internal standard; LA, linoleic acid

Table S3. Repeatability of urinary lipid extraction procedure using three SPE products

Description	Strata-X		Oasis PRiME HLB		Monospin C18	
	mean ± SD	CV	mean ± SD	CV	mean ± SD	CV
tetranor-PGEM	7.12 ± 0.61	8.6	6.51 ± 0.10	1.5	6.51 ± 0.28	4.3
tetranor-PGDM	6.53 ± 0.53	8.2	5.93 ± 0.22	3.6	7.20 ± 0.24	3.3
6-keto-PGF1α	4.57 ± 0.20	4.3	4.44 ± 0.13	2.8	4.64 ± 0.16	3.4
TXB2	26.05 ± 1.15	4.4	25.76 ± 0.35	1.4	25.12 ± 0.80	3.2
8-iso-PGF2α	1.57 ± 0.04	2.4	1.54 ± 0.11	6.9	1.50 ± 0.01	0.6
PGE2	1.82 ± 0.16	8.7	1.72 ± 0.09	5.2	1.82 ± 0.02	1.1
PGD2	2.60 ± 0.09	3.6	2.71 ± 0.03	0.9	2.68 ± 0.07	2.6
15-keto-PGE2	0.67 ± 0.02	3.4	0.69 ± 0.01	1.2	0.72 ± 0.03	4.3
PGF2α	1.89 ± 0.10	5.0	1.78 ± 0.11	5.9	1.86 ± 0.07	3.8
11-dehydro-TXB2	0.39 ± 0.04	9.3	0.38 ± 0.01	2.6	0.42 ± 0.01	2.9
PGA2	0.38 ± 0.03	8.9	0.40 ± 0.02	3.9	0.41 ± 0.02	3.8
LTB4	10.49 ± 0.54	5.1	8.99 ± 0.91	10.2	11.26 ± 0.87	7.7
LTD4	0.58 ± 0.02	2.7	0.65 ± 0.03	5.1	0.61 ± 0.02	2.9
15-HETE	1.17 ± 0.17	14.2	1.23 ± 0.11	8.8	1.30 ± 0.11	8.3
12-HETE	2.97 ± 0.52	17.6	2.83 ± 0.20	7.0	3.11 ± 0.29	9.2
5-HETE	2.72 ± 0.36	13.1	2.53 ± 0.43	16.8	2.90 ± 0.16	5.6
11,12-EET	0.64 ± 0.08	13.1	0.83 ± 0.14	16.9	0.83 ± 0.08	9.9
PAF	2.17 ± 0.12	5.5	1.35 ± 0.82	60.3	2.23 ± 0.06	2.6
AA	7.58 ± 0.76	10.0	6.94 ± 0.09	1.3	7.51 ± 0.13	1.8

Data are shown as mean ± SD of the peak area ratio to internal standard and CV [%] (n = 3).

Table S4. Diurnal fluctuation of urinary excretion of lipid metabolites

		peak arearatio of 24-hour urine	Sampling time<br>8-12	12-14	14-17	17-24	24-6	6-8	average gap to 24-hour urine	coefficient of variation
			upper: the ratio of measured values to the values in 24-hours urine<br>lower: the ratio of creatinine normalized values to the values in 24-hours urine							
tetranor-PGEM	A	2.61	1.08	0.90	1.07	1.38	1.46	2.50	1.40	0.41
			0.54	0.49	0.52	0.56	0.58	0.68	0.56	0.11
	B	0.58	1.77	2.36	1.61	2.97	2.10	1.64	2.07	0.25
			2.44	1.95	1.93	1.72	1.42	1.07	1.75	0.27
	C	2.73	0.79	2.80		1.65	0.99	0.76	1.40	0.62
			1.17	1.26		0.75	0.50	1.01	0.94	0.33
	D	0.99	2.48	1.86	1.42	0.91	0.94	2.01	1.61	0.39
			0.98	0.82	0.78	0.65	0.79	1.07	0.85	0.18
	E	0.37	3.84	4.26		5.04	0.99	0.76	2.98	0.66
			2.38	4.79		2.25	0.46	0.32	2.04	0.89
tetranor-PGDM	A	0.85	0.97	0.84	1.11	1.33	1.71	1.91	1.31	0.32
			0.48	0.46	0.54	0.54	0.68	0.52	0.54	0.14
	B	0.58	1.42	2.77	2.48	2.84	2.66	1.21	2.23	0.32
			1.96	2.28	2.99	1.64	1.79	0.79	1.91	0.38
	C	0.94	0.34	1.54		1.50	1.32	0.85	1.11	0.46
			0.51	0.70		0.69	0.66	1.13	0.74	0.32
	D	1.00	1.30	1.15	0.92	0.80	0.99	2.05	1.20	0.38
			0.51	0.50	0.50	0.57	0.83	1.10	0.67	0.36
	E	0.22	11.95	6.90		7.33	2.76	2.00	6.19	0.65
			7.40	7.76		3.27	1.29	0.85	4.11	0.80
tetranor-PGAM	A	0.16	1.58	1.26	1.26	1.48	1.15	1.60	1.39	0.14
			0.79	0.69	0.61	0.60	0.45	0.43	0.60	0.23
	B	0.12	1.02	1.62	1.20	2.03	2.47	2.52	1.81	0.35
			1.40	1.34	1.45	1.18	1.67	1.65	1.45	0.13
	C	0.12	0.84	1.66		1.57	1.64	0.65	1.27	0.38
			1.25	0.75		0.72	0.82	0.86	0.88	0.24
	D	0.14	2.19	1.78	1.39	0.97	0.52	0.98	1.31	0.47

			0.86	0.78	0.76	0.69	0.43	0.52	0.68	0.24
	E	0.14	2.91	1.51		1.51	1.51	1.18	1.72	0.39
			1.80	1.69		0.68	0.71	0.50	1.08	0.58
13,14-dihydro-15-keto-PGF1α	A	0.17	1.16	1.12	0.81	0.79	0.95	0.96	0.97	0.16
			0.58	0.62	0.40	0.32	0.37	0.26	0.42	0.34
	B	0.28	2.53	2.04	0.90	0.90	0.88	0.99	1.37	0.53
			3.50	1.68	1.08	0.52	0.59	0.65	1.34	0.86
	C	0.19	0.58	2.26		0.70	1.94	0.52	1.20	0.70
			0.86	1.02		0.32	0.97	0.68	0.77	0.37
	D	0.42	0.93	0.41	0.35	0.71	2.15	1.21	0.96	0.69
			0.36	0.18	0.19	0.51	1.79	0.65	0.61	0.99
	E	0.60	2.55	1.59		2.42	1.91	1.01	1.90	0.33
			1.58	1.78		1.08	0.89	0.43	1.15	0.47
13,14-dihydro-15-keto-tetranor-PGD2	A	0.44	0.89	0.79	1.29	1.47	1.54	2.54	1.42	0.44
			0.44	0.43	0.63	0.60	0.61	0.69	0.57	0.18
	B	not detected								
	C	0.61	0.24	1.91		1.00	0.92	0.29	0.87	0.78
			0.35	0.86		0.46	0.46	0.39	0.50	0.41
	D	0.52	1.96	1.13	0.85	0.59	0.77	1.80	1.18	0.48
			0.77	0.50	0.46	0.42	0.64	0.96	0.63	0.33
	E	0.27	1.61	4.72		4.06	2.62	2.54	3.11	0.40
			1.00	5.30		1.81	1.22	1.08	2.08	0.88
13,14-dihydro-15-keto-tetranor-PGE2	A	1.81	0.62	0.58	0.73	1.19	2.23	3.41	1.46	0.78
			0.31	0.32	0.36	0.48	0.88	0.92	0.55	0.52
	B	0.46	0.78	2.27	1.56	2.56	2.55	1.43	1.86	0.39
			1.07	1.87	1.87	1.48	1.72	0.93	1.49	0.27
	C	3.48	0.18	1.26		1.66	1.70	0.68	1.10	0.60
			0.26	0.57		0.76	0.85	0.90	0.67	0.39
	D	2.69	1.37	1.01	0.76	0.63	0.80	1.79	1.06	0.42
			0.54	0.45	0.41	0.45	0.67	0.95	0.58	0.36
	E	0.54	2.78	3.91		3.62	5.00	5.26	4.12	0.25
			1.72	4.40		1.61	2.34	2.24	2.46	0.46
PGF2α	A	0.06	0.69	0.55	0.00	0.89	0.00	1.23	0.56	0.87

			0.35	0.30	0.00	0.36	0.00	0.33	0.22	0.78
	B	0.04	0.00	0.00	1.13	1.52	1.70	1.34	0.95	0.80
			0.00	0.00	1.35	0.88	1.14	0.87	0.71	0.81
	C	0.06	0.41	1.90		1.87	1.36	0.56	1.22	0.58
			0.61	0.86		0.85	0.68	0.74	0.75	0.14
	D	0.04	2.28	1.35	1.80	1.26	1.00	2.10	1.63	0.31
			0.90	0.59	0.98	0.90	0.83	1.12	0.89	0.20
	E	0.05	2.60	2.35		1.42	2.12	1.48	2.00	0.26
			1.61	2.65		0.64	0.99	0.63	1.30	0.65
PGF1α	A	0.05	0.63	0.75	0.34	0.73	1.14	1.02	0.77	0.37
			0.31	0.41	0.17	0.30	0.45	0.28	0.32	0.32
	B	0.05	1.35	1.24	1.51	0.92	1.14	0.78	1.16	0.24
			1.86	1.02	1.82	0.53	0.77	0.51	1.09	0.57
	C	0.04	1.13	1.05		1.01	1.23	1.22	1.13	0.09
			1.68	0.47		0.46	0.62	1.62	0.97	0.64
	D	0.03	2.19	1.00	1.36	1.90	1.44	0.81	1.45	0.36
			0.86	0.44	0.74	1.36	1.20	0.43	0.84	0.46
	E	0.03	1.67	1.17		1.58	1.52	2.85	1.76	0.36
			1.03	1.32		0.70	0.71	1.21	0.99	0.28
15-keto-PGE2	A	0.22	2.49	1.93	2.06	0.91	0.59	0.49	1.41	0.60
			1.24	1.06	1.00	0.37	0.23	0.13	0.67	0.72
	B	0.01	0.58	0.75	0.93	2.83	2.07	2.13	1.55	0.59
			0.80	0.62	1.12	1.64	1.39	1.39	1.16	0.34
	C	0.02	0.00	0.00		3.70	1.58	0.32	1.12	1.41
			0.00	0.00		1.69	0.79	0.42	0.58	1.21
	D	0.10	1.81	0.96	0.53	0.56	1.58	1.58	1.17	0.48
			0.71	0.42	0.29	0.40	1.32	0.85	0.66	0.58
	E	0.03	2.73	2.47		2.47	4.27	4.23	3.23	0.29
			1.69	2.78		1.10	1.99	1.80	1.87	0.32
LTB4-EA	A	0.03	0.57	1.34	1.60	1.23	1.17	1.29	1.20	0.29
			0.28	0.74	0.78	0.50	0.46	0.35	0.52	0.39
	B	0.02	2.21	2.45	1.67	2.82	2.61	2.23	2.33	0.17
			3.05	2.02	2.01	1.63	1.76	1.46	1.99	0.28
	C	0.02	0.62	0.87		1.97	1.47	0.66	1.12	0.53

			0.92	0.39		0.90	0.74	0.87	0.76	0.29
	D	0.02	3.04	2.17	1.51	0.93	0.61	1.25	1.58	0.56
			1.19	0.95	0.82	0.67	0.50	0.67	0.80	0.31
	E	0.01	2.88	3.37		3.29	5.23	1.81	3.32	0.37
			1.79	3.78		1.47	2.44	0.77	2.05	0.56
Maresin1	A	0.31	0.93	0.80	0.55	0.60	0.91	2.75	1.09	0.76
			0.46	0.44	0.27	0.24	0.36	0.75	0.42	0.44
	B	0.08	0.00	0.00	0.00	0.00	2.52	1.98	0.75	1.57
			0.00	0.00	0.00	0.00	1.70	1.30	0.50	1.57
	C	0.12	0.00	0.00		0.00	0.00	0.71		
			0.00	0.00		0.00	0.00	0.94		
	D	0.49	1.12	2.77	2.22	1.23	0.53	1.35	1.53	0.53
			0.44	1.21	1.21	0.88	0.44	0.72	0.82	0.43
	E	0.28	0.99	2.17		3.84	3.52	2.30	2.56	0.45
			0.62	2.44		1.71	1.64	0.98	1.48	0.48
9,10-DiHOME	A	0.09	1.22	0.79	0.22	2.35	1.76	0.64	1.16	0.67
			0.61	0.44	0.11	0.95	0.69	0.17	0.49	0.65
	B	0.05	3.66	1.06	0.49	0.37	1.24	0.54	1.23	1.01
			5.05	0.88	0.59	0.21	0.84	0.35	1.32	1.40
	C	0.01	0.22	1.76		0.39	3.56	0.46	1.28	1.11
			0.33	0.79		0.18	1.79	0.61	0.74	0.86
	D	0.05	5.18	0.60	1.12	1.12	2.59	1.31	1.99	0.86
			2.04	0.26	0.61	0.80	2.16	0.70	1.10	0.73
	E	0.05	6.98	5.69		6.68	2.16	2.36	4.77	0.49
			4.33	6.39		2.98	1.01	1.00	3.14	0.73
Lyso-PAF	A	0.58	1.88	1.60	1.59	1.49	1.39	1.43	1.56	0.11
			0.94	0.88	0.77	0.60	0.55	0.39	0.69	0.31
	B	0.26	1.76	2.49	2.24	2.31	2.22	2.01	2.17	0.12
			2.43	2.06	2.69	1.34	1.49	1.31	1.89	0.31
	C	0.31	1.33	1.13		1.40	1.55	1.11	1.30	0.14
			1.98	0.51		0.64	0.78	1.47	1.07	0.58
	D	0.33	1.08	1.37	0.96	0.89	0.93	0.85	1.01	0.19
			0.43	0.60	0.53	0.63	0.78	0.46	0.57	0.23
	E	0.16	3.87	1.90		2.05	1.86	1.53	2.24	0.42

			2.40	2.13		0.92	0.87	0.65	1.39	0.58
OEA	A	0.006	2.93	1.31	4.93	1.15	2.19	1.65	2.36	0.60
			1.46	0.72	2.40	0.47	0.86	0.45	1.06	0.71
	B	0.003	1.50	3.92	2.71	1.98	6.80	2.72	3.27	0.59
			2.07	3.23	3.26	1.15	4.58	1.78	2.68	0.47
	C	0.006	0.76	1.29		0.79	1.31	5.65	1.96	1.06
			1.13	0.58		0.36	0.66	7.49	2.04	1.49
	D	0.051	0.18	0.15	0.35	0.42	3.74	1.20	1.01	1.38
			0.07	0.06	0.19	0.30	3.12	0.64	0.73	1.62
	E	0.034	0.30	0.16		0.31	0.19	0.12	0.22	0.39
			0.18	0.18		0.14	0.09	0.05	0.13	0.45
DHA	A	0.03	1.93	1.50	1.24	1.41	2.96	1.16	1.70	0.40
			0.96	0.82	0.60	0.57	1.17	0.31	0.74	0.41
	B	0.03	1.20	0.87	0.00	1.25	0.74	0.00	0.68	0.83
			1.66	0.72	0.00	0.72	0.50	0.00	0.60	1.02
	C	0.02	0.74	0.73		2.49	2.46	1.30	1.54	0.57
			1.10	0.33		1.14	1.24	1.72	1.10	0.45
	D	0.03	1.11	0.79	0.96	1.58	1.64	1.94	1.34	0.34
			0.43	0.35	0.52	1.13	1.37	1.04	0.81	0.53
	E	0.02	1.23	1.41		1.25	2.24	1.45	1.51	0.27
			0.76	1.58		0.56	1.04	0.62	0.91	0.46

Data are shown as peak area ratio in 24-hour urine (/day), the ratio of measured values to the values in 24-hour urine (upper row) and the ratio of creatinine normalized values to the values in 24-hour urine (lower row).

Table S5. Stratified analysis for the metabolites by RAS inhibitors

		non-DM	stage 1	stage 2	stage 3-4
n	yes	0	14	20	17
	no	33	37	19	4
tetranor-PGEM	yes	-	17.17 [11.20, 41.17]	31.79 [20.45, 44.87]	42.61 [34.79, 52.67] †
	no	12.51 [7.96, 17.75]	19.03 [10.33, 28.33]*	27.58 [23.76, 41.90]*	31.69 [24.51, 37.36]*
13,14-dihydro-15-keto-tetranor-PGE2	yes	-	7.69 [4.47, 11.50]	10.42 [5.87, 15.33]	7.09 [2.40, 15.06]
	no	13.06 [9.45, 28.31]	5.70 [3.24, 12.59]*	7.36 [5.02, 11.55]*	7.92 [5.14, 10.00]
PGE2	yes	-	0.12 [0.00, 0.17]	0.10 [0.00, 0.20]	0.17 [0.10, 0.40]
	no	0.00 [0.00, 0.00]	0.06 [0.00, 0.12]*	0.08 [0.00, 0.15]	0.14 [0.09, 0.26]
13,14-dihydro-15-keto-PGE2	yes	-	0.00 [0.00, 0.03]	0.04 [0.00, 0.09]	0.10 [0.00, 0.16] †
	no	0.00 [0.00, 0.00]	0.00 [0.00, 0.04]*	0.00 [0.00, 0.06]*	0.04 [0.04, 0.05]*
13,14-dihydro-15-keto-PGA2	yes	-	0.00 [0.00, 0.00]	0.00 [0.00, 0.00]	0.00 [0.00, 0.53] †
	no	0.00 [0.00, 0.00]	0.00 [0.00, 0.00]	0.00 [0.00, 0.00]*	0.00 [0.00, 0.16]*
8-iso-PGE2	yes	-	0.00 [0.00, 0.07]	0.00 [0.00, 0.00]	0.00 [0.00, 0.03]
	no	0.00 [0.00, 0.00]	0.00 [0.00, 0.03]*	0.00 [0.00, 0.00]	0.00 [0.00, 0.01]*
5-iPF2α-VI	yes	-	0.47 [0.29, 0.67]	0.38 [0.25, 0.57]	0.16 [0.00, 0.40] † ‡

	no	0.53 [0.42, 0.77]	0.50 [0.30, 0.76]	0.47 [0.31, 0.64]	0.05 [0.00, 0.26]
11-dehydro-TXB2	yes	-	0.16 [0.02, 0.24]	0.19 [0.13, 0.24]	0.08 [0.07, 0.21]
	no	0.14 [0.08, 0.22]	0.17 [0.12, 0.26]	0.24 [0.13, 0.41]	0.11 [0.09, 0.14]
2,3-dinor-TXB2	yes	-	0.21 [0.13, 0.43]	0.63 [0.08, 3.16]	0.05 [0.00, 0.11] ‡
	no	0.12 [0.06, 0.44]	0.14 [0.00, 0.71]	0.26 [0.05, 0.74]	0.09 [0.00, 0.18]
PGD2	yes	-	0.11 [0.00, 0.17]	0.04 [0.00, 0.15]	0.16 [0.00, 0.24]
	no	0.00 [0.00, 0.00]	0.07 [0.00, 0.25]	0.00 [0.00, 0.20]	0.11 [0.08, 0.20]
LTB4-EA	yes	-	0.18 [0.09, 0.32]	0.24 [0.08, 0.38]	0.20 [0.07, 0.25]
	no	0.32 [0.17, 0.48]	0.31 [0.15, 0.53]	0.31 [0.20, 0.52]	0.08 [0.05, 0.16]
Maresin1	yes	-	11.01 [7.83, 21.48]	16.44 [7.75, 24.87]	2.78 [0.83, 7.13] † ‡
	no	11.50 [6.27, 20.71]	10.89 [3.75, 21.00]	11.06 [6.69, 20.53]	2.01 [0.83, 6.81]
9,10-DiHOME	yes	-	0.20 [0.09, 0.30]	0.54 [0.31, 0.77] †	0.22 [0.16, 0.87]
	no	0.47 [0.16, 1.19]	0.12 [0.03, 0.36]*	0.47 [0.19, 0.77] †	0.43 [0.20, 0.64]
Lyso-PAF	yes	-	0.09 [0.06, 0.14]	0.21 [0.15, 0.30] †	0.84 [0.52, 1.33] † ‡
	no	0.16 [0.07, 0.24]	0.12 [0.08, 0.20]	0.20 [0.12, 0.66]	0.42 [0.30, 0.71]

Data are expressed as median (ng/mgCr) and interquartile range [Q1, Q3], (n). *: $P < 0.05$ vs. non-DM, †: $P < 0.05$ vs. stage 1, ‡: $P < 0.05$ vs. stage 2, a: $P < 0.05$ vs. “yes”.

Table S6. Stratified analysis for the metabolites by platelet inhibitors

		non-DM	stage 1	stage 2	stage 3-4
n	yes	1	7	12	9
	no	32	44	27	12
tetranor-PGEM	yes	1.76 [NA]	19.90 [7.89, 44.74]	24.07 [19.28, 42.26]	36.59 [30.36, 48.29]
	no	12.79 [8.17, 17.87]	19.00 [10.86, 31.59]*	30.44 [24.13, 43.23]* †	40.81 [36.30, 51.74]* †
13,14-dihydro-15-keto-tetranor-PGE2	yes	4.35 [4.35, 4.35]	5.16 [3.77, 14.53]	6.50 [3.73, 12.49]	2.40 [1.73, 15.06]
	no	13.46 [9.66, 28.49]	5.90 [3.86, 11.82]*	9.43 [5.84, 14.29]	8.51 [5.74, 10.31]
PGE2	yes	0.30 [0.30, 0.30]	0.13 [0.06, 0.19]	0.05 [0.00, 0.13]	0.15 [0.12, 0.21]
	no	0.00 [0.00, 0.00]	0.06 [0.00, 0.13]*	0.09 [0.00, 0.20]*	0.17 [0.09, 0.43]* †
13,14-dihydro-15-keto-PGE2	yes	0.00 [0.00, 0.00]	0.00 [0.00, 0.02]	0.03 [0.00, 0.08]	0.04 [0.00, 0.06]
	no	0.00 [0.00, 0.00]	0.00 [0.00, 0.04]*	0.00 [0.00, 0.09]*	0.10 [0.04, 0.13]* †
13,14-dihydro-15-keto-PGA2	yes	0.00 [0.00, 0.00]	0.00 [0.00, 0.00]	0.00 [0.00, 0.09]	0.00 [0.00, 0.49]
	no	0.00 [0.00, 0.00]	0.00 [0.00, 0.00]	0.00 [0.00, 0.00]	0.00 [0.00, 0.57]*
8-iso-PGE2	yes	0.00 [0.00, 0.00]	0.06 [0.00, 0.09]	0.00 [0.00, 0.00]	0.00 [0.00, 0.03]
	no	0.00 [0.00, 0.00]	0.00 [0.00, 0.01]	0.00 [0.00, 0.00]	0.00 [0.00, 0.00]
5-iPF2α-VI	yes	0.90 [0.90, 0.90]	0.52 [0.29, 0.65]	0.26 [0.21, 0.36]	0.11 [0.00, 0.16] †

	no	0.52 [0.41, 0.75]	0.48 [0.30, 0.76]	0.48 [0.37, 0.63] [a]	0.31 [0.00, 0.52]
11-dehydro-TXB2	yes	0.00 [0.00, 0.00]	0.08 [0.00, 0.16]	0.15 [0.09, 0.20]	0.07 [0.00, 0.08] [‡]
	no	0.15 [0.10, 0.22]	0.18 [0.12, 0.25]	0.24 [0.16, 0.39]* [a]	0.17 [0.13, 0.25] [a]
2,3-dinor-TXB2	yes	0.00 [0.00, 0.00]	0.13 [0.00, 0.59]	0.34 [0.08, 0.67]	0.00 [0.00, 0.08]
	no	0.13 [0.06, 0.46]	0.19 [0.05, 0.61]	0.51 [0.08, 3.75]	0.08 [0.00, 0.24]
PGD2	yes	0.00 [0.00, 0.00]	0.08 [0.00, 0.14]	0.13 [0.00, 0.16]	0.16 [0.12, 0.31]
	no	0.00 [0.00, 0.00]	0.10 [0.00, 0.25]	0.00 [0.00, 0.17]	0.13 [0.00, 0.22]
LTB4-EA	yes	0.39 [0.39, 0.39]	0.25 [0.18, 0.49]	0.21 [0.03, 0.66]	0.07 [0.00, 0.14]
	no	0.31 [0.17, 0.48]	0.28 [0.12, 0.47]	0.30 [0.20, 0.39]	0.22 [0.09, 0.30]
Maresin1	yes	11.28 [11.28, 11.28]	13.88 [8.41, 27.43]	18.70 [5.61, 22.13]	0.83 [0.56, 1.43] [† ‡]
	no	12.05 [6.06, 20.93]	10.51 [5.16, 18.46]	11.06 [7.71, 24.02]	6.88 [3.36, 10.70] [a]
9,10-DiHOME	yes	0.47 [0.47, 0.47]	0.17 [0.06, 0.27]	0.56 [0.53, 0.81] [†]	0.28 [0.15, 0.64]
	no	0.43 [0.16, 1.21]	0.14 [0.04, 0.32]*	0.46 [0.19, 0.73] [†]	0.22 [0.18, 1.08]
Lyso-PAF	yes	0.38 [0.38, 0.38]	0.10 [0.06, 0.13]	0.17 [0.14, 0.26]	0.76 [0.48, 0.91] [‡]
	no	0.15 [0.07, 0.22]	0.12 [0.08, 0.20]	0.23 [0.13, 0.58] [†]	0.83 [0.40, 1.63]* [†]

Data are expressed as median (ng/mgCr) and interquartile range [Q1, Q3], (n). *: $P < 0.05$ vs. non-DM, † : $P < 0.05$ vs. stage 1, ‡ : $P < 0.05$ vs. stage 2, a: $P < 0.05$ vs. “yes”.

Fig. S1

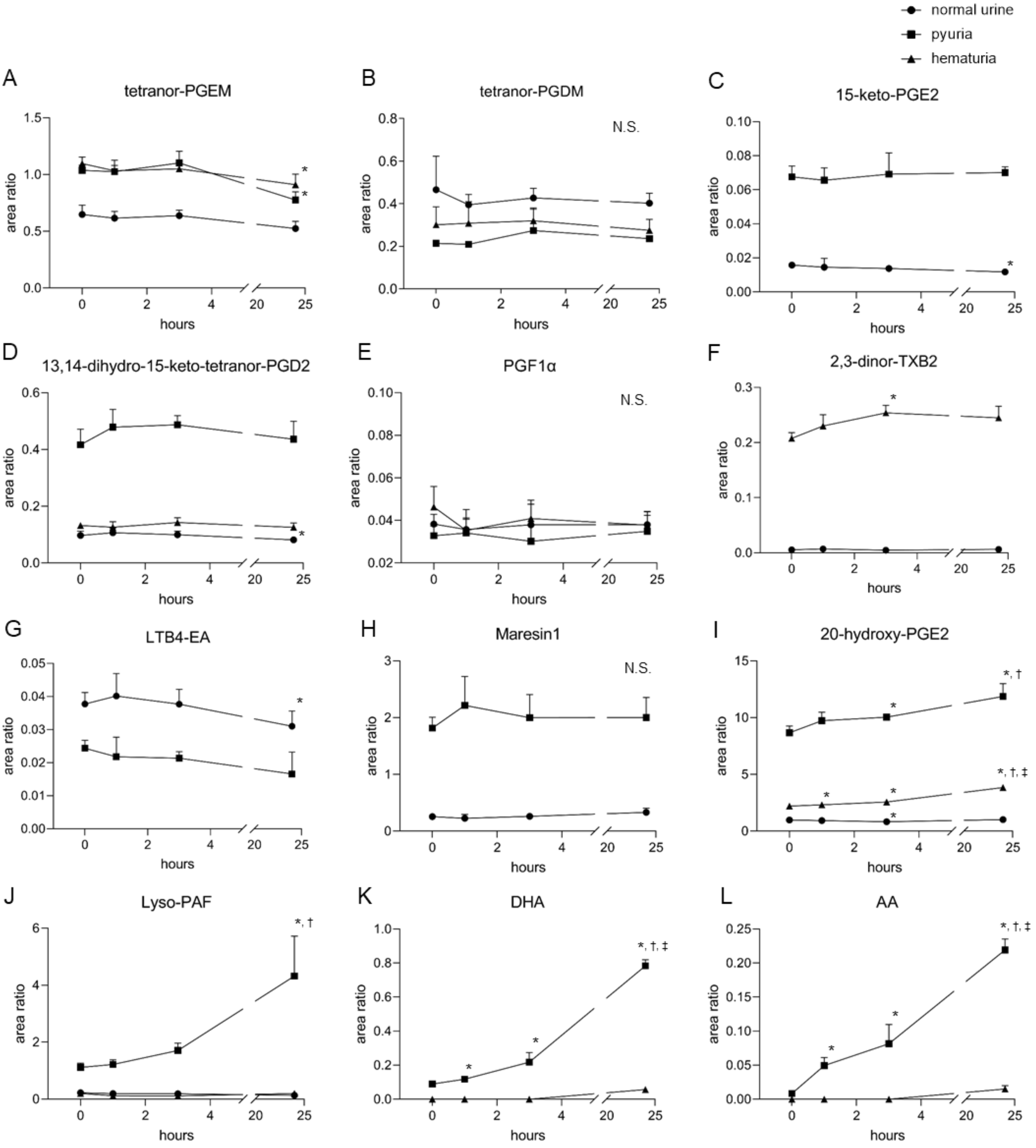

Figure S1. Effects of incubation on urinary eicosanoids and related mediators. We investigated the effects of incubation on the measurement of urinary eicosanoids and related mediators using three samples: normal (closed circle), pyuria (closed square), and hematuria (closed triangle). Each sample was incubated at 0, 1, 3, and 24 hours; the metabolites that were detected in the urinary samples are shown. Data are shown as the mean ± SD of the peak area ratio (n = 5); *$P < 0.05$ versus 0 h, $^{\dagger}P < 0.05$ versus 1 h, $^{\ddagger}P < 0.05$ versus 3 h.

Fig. S2

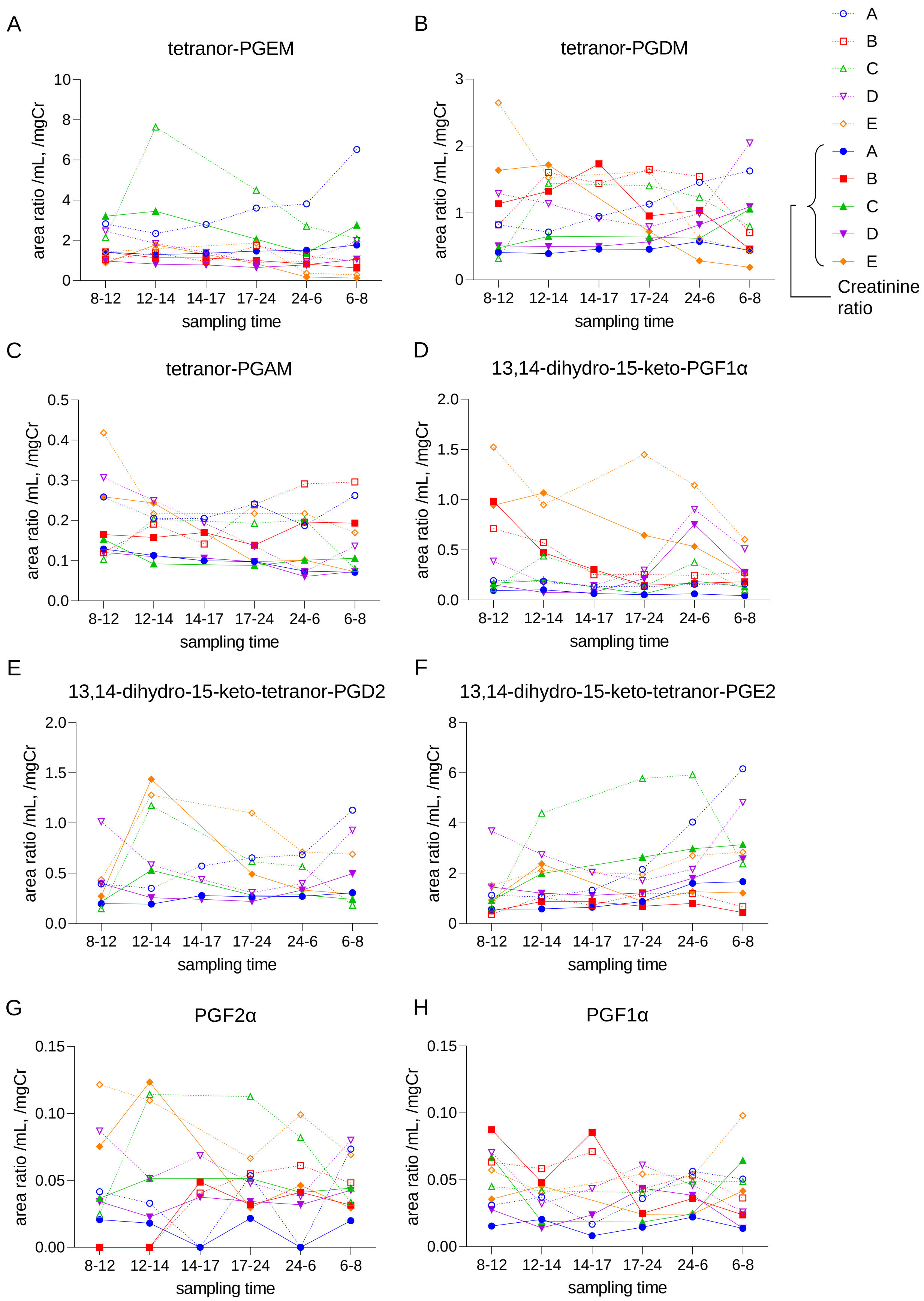
A
tetranor-PGEM
B
tetranor-PGDM
C
tetranor-PGAM
D
13,14-dihydro-15-keto-PGF1α
E
13,14-dihydro-15-keto-tetranor-PGD2
F
13,14-dihydro-15-keto-tetranor-PGE2
G
PGF2α
H
PGF1α
area ratio /mL, /mgCr
sampling time
8-12
12-14
14-17
17-24
24-6
6-8
A
B
C
D
E
A
B
C
D
E
Creatinine ratio

Fig. S2 (continued)

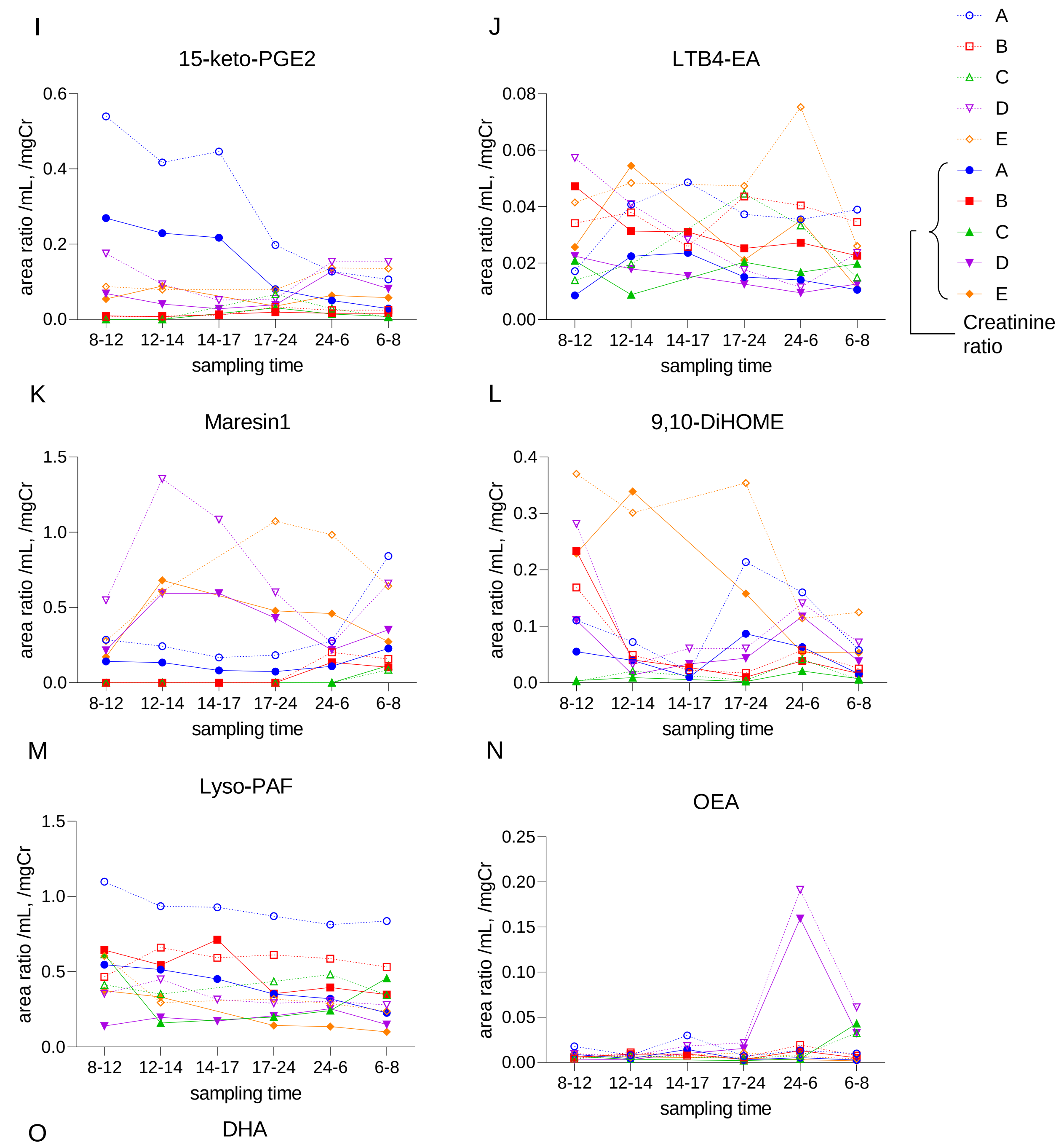

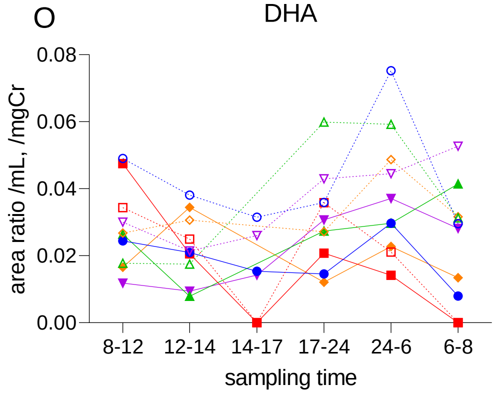

Figure S2. Diurnal fluctuation of urinary excretion of lipid metabolites. We investigated the diurnal fluctuation of the urinary excretion of lipid metabolites using spot urine and 24-hour urine specimens collected from healthy volunteers (n = 5). The measured values (open symbol) and the values of the ratio to urinary creatinine (closed symbol) are shown. Data are shown as the peak area ratio.

Fig. S3

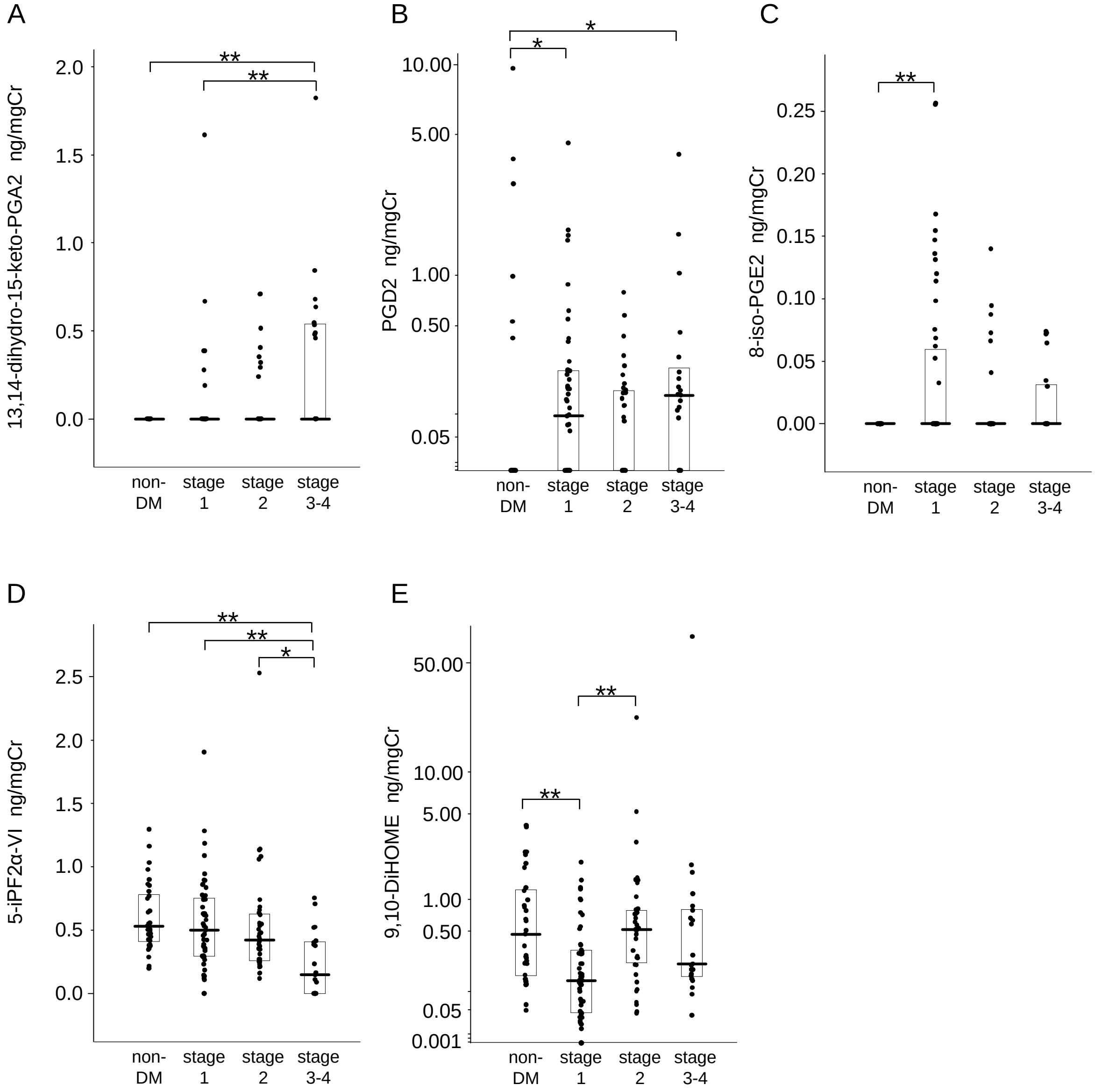

Figure S3. Modulation of urinary eicosanoids and related mediators according to the stages of diabetic nephropathy. We compared the urinary lipid metabolite levels between the subjects with diabetes and the control group. (A) 13,14-dihydro-15-keto-prostaglandin A2 (13,14-dihyro-15-keto-PGA2); (B) prostaglandin D2 (PGD2); (C) 8-iso-PGE2; (D)5-isoprostane F2α-VI (5-iPF2α-VI); (E) 9,10-dihydroxy-12Z-octadecenoic acid (9,10-DiHOME). Data are shown as the median ± SE, **P* < 0.05, ***P* < 0.01.

Fig. S4

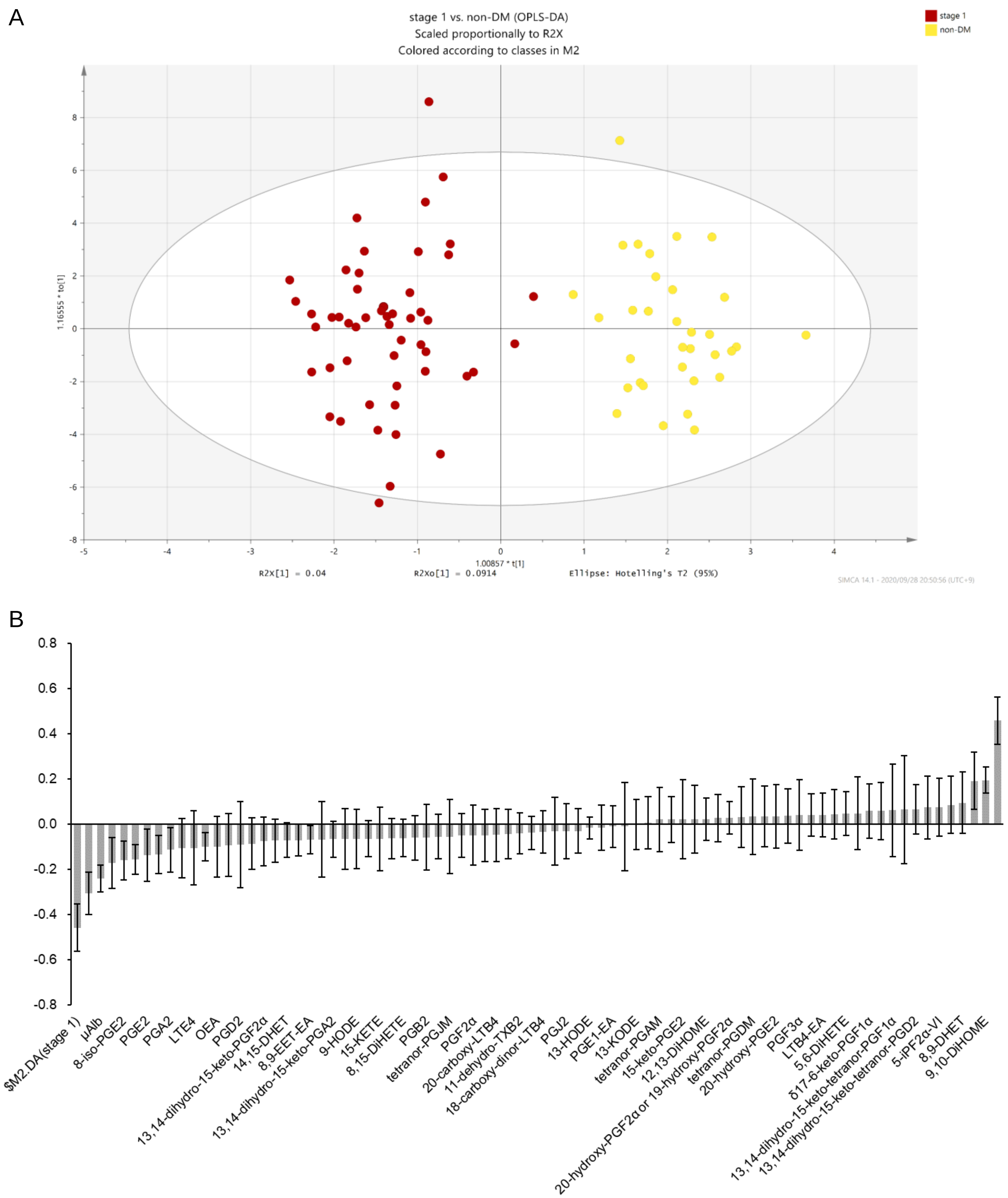

Figure S4. OPLS analysis for metabolites with variable importance in projection in stage 1 nephropathy group versus control group. Score scatter plot (A) and loading column plot (B) between stage 1 nephropathy group (closed red circle) and control group (closed yellow circle) were shown.

Fig. S5

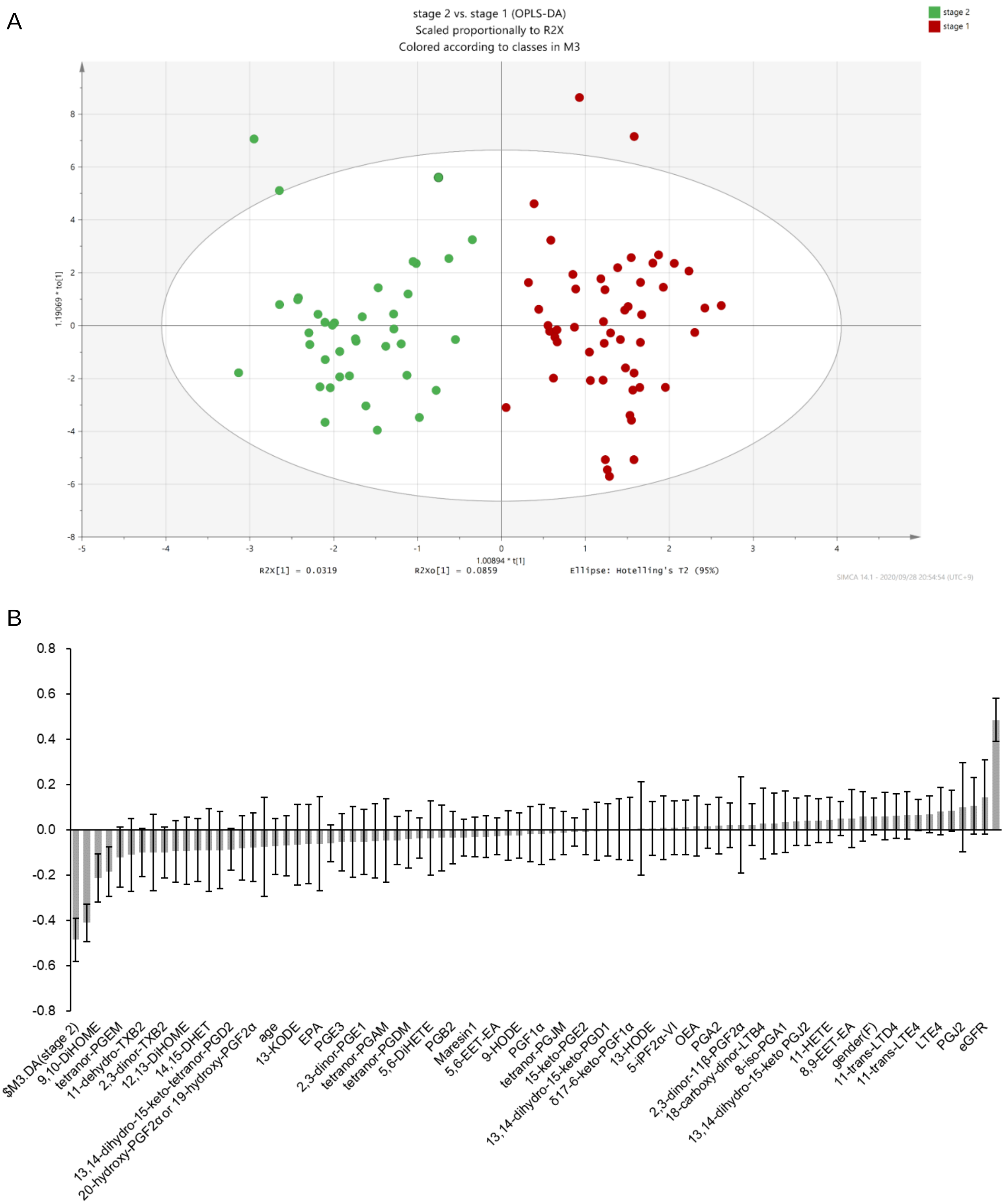

Figure S5. OPLS analysis for metabolites with variable importance in projection in stage 2 nephropathy group versus stage 1 nephropathy group. Score scatter plot (A) and loading column plot (B) between stage 2 nephropathy group (closed green circle) and stage 1 nephropathy group (closed red circle) were shown.

Fig. S6

A

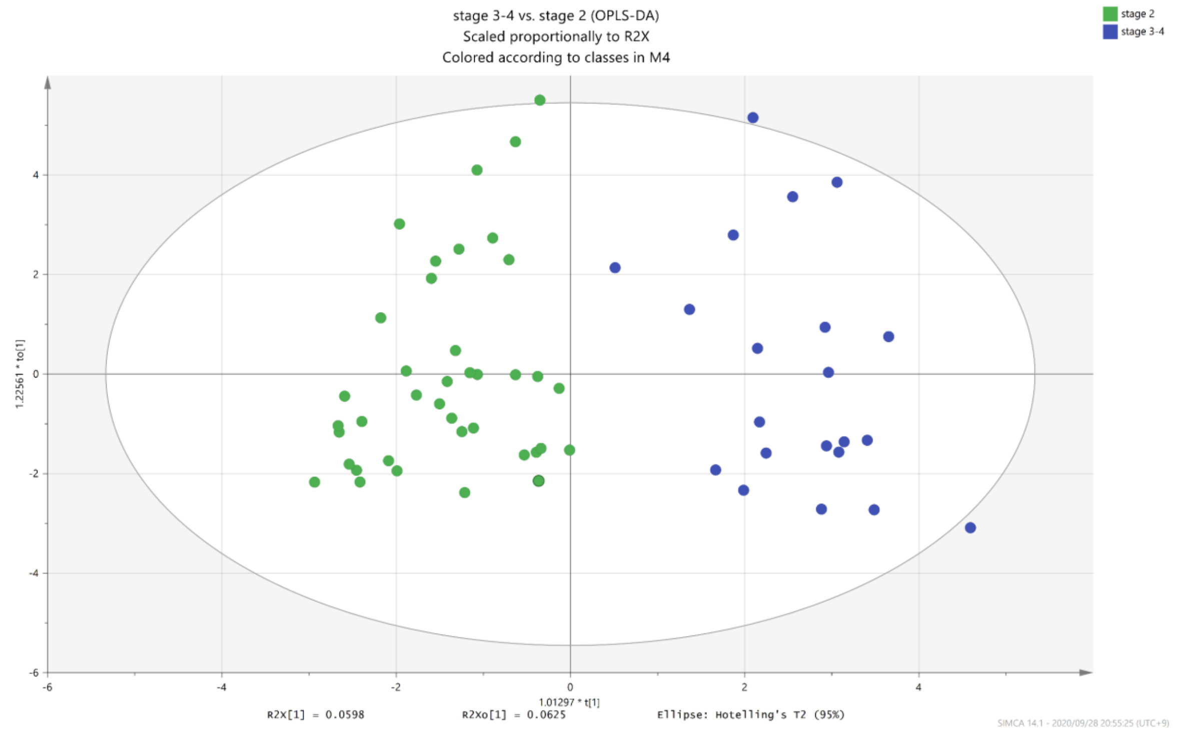

B

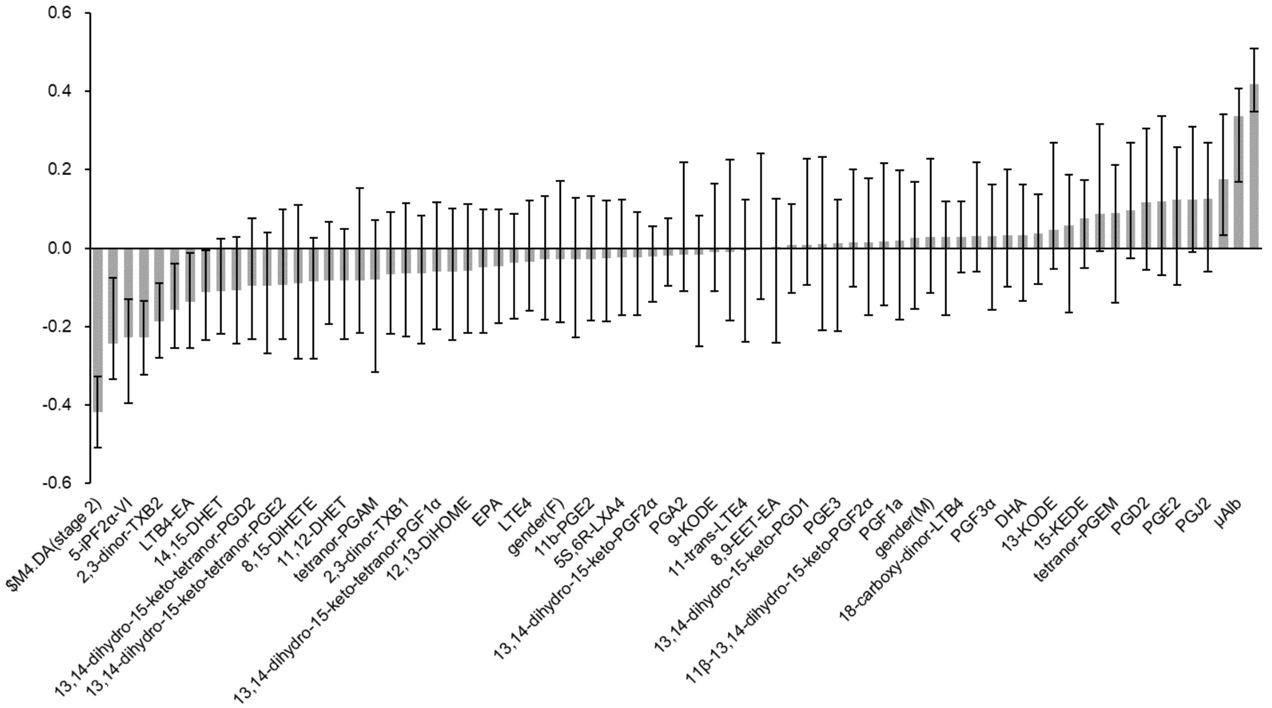

Figure S6. OPLS analysis for metabolites with variable importance in projection in stage 3-4 nephropathy group versus stage 2 nephropathy group. Score scatter plot (A) and loading column plot (B) between stage 3-4 nephropathy group (closed blue circle) and stage 2 nephropathy group (closed green circle) were shown.

Fig. S7

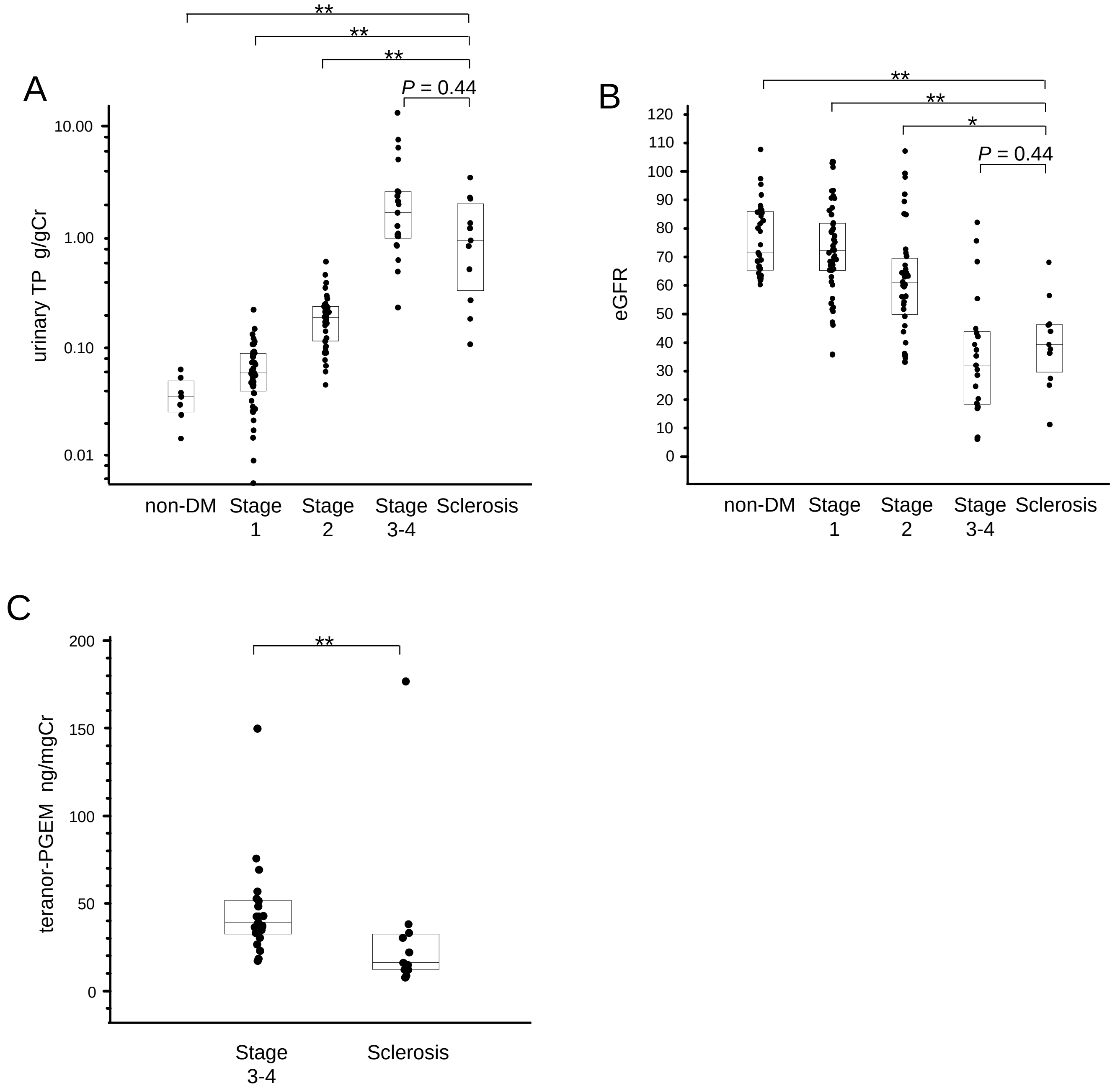

Figure S7. Comparison of the urinary tetranor-PGEM in subjects with diabetic nephropathy and renal sclerosis. We compared the levels of urinary TP (A) and eGFR (B) among the subjects with diabetic nephropathy and renal sclerosis. (C) The levels of urinary tetranor-PGEM were compared between the stage 3-4 nephropathy and sclerosis. Data are shown as the median ± SE, *$P < 0.05$, **$P < 0.01$.

Fig. S8

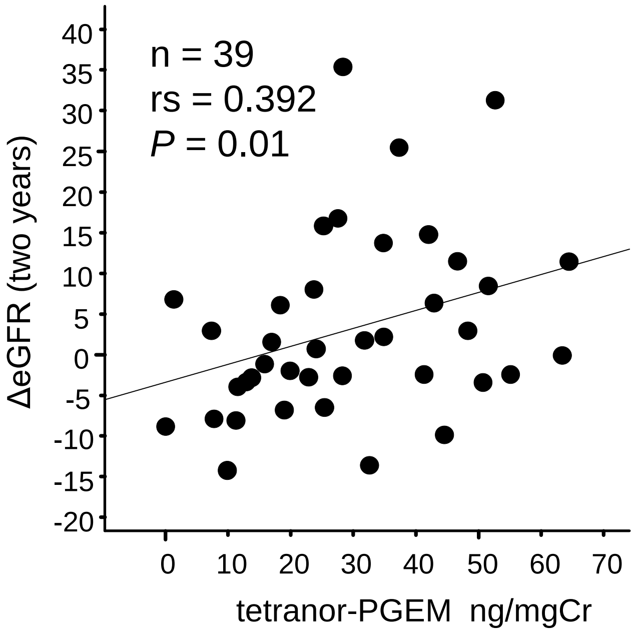

Figure S8. Association between urinary tetranor-PGEM and the decline in eGFR level. Correlation analyses between urinary tetranor-PGEM and the decline in eGFR level for two years were performed. The Spearman's rank correlation coefficient is shown.

Fig. S9

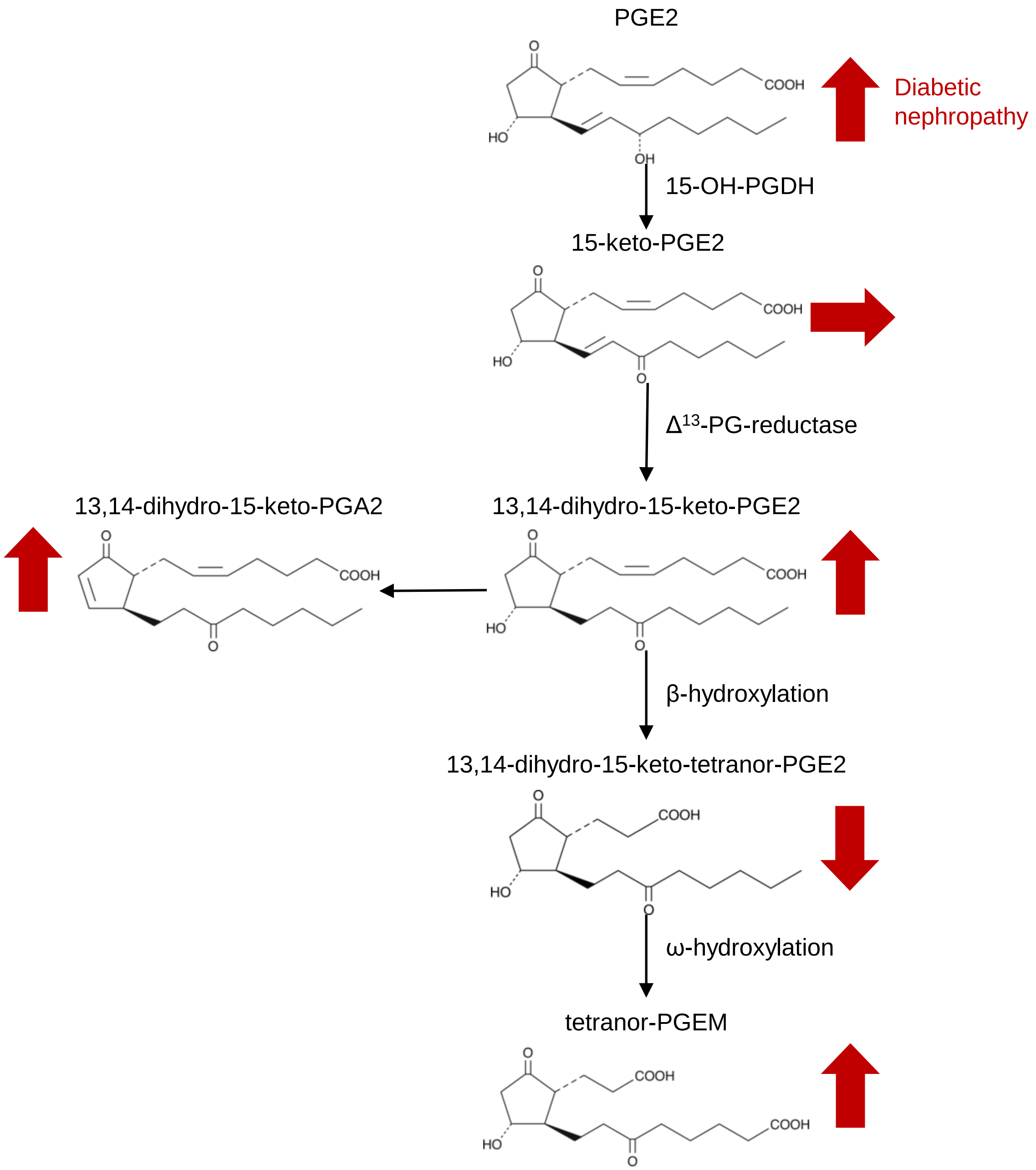

Figure S9. Schematic figure of prostaglandin E metabolites pathway. The metabolic pathway of prostaglandin E (PGE) were shown. Red arrows show the modulation of urinary PGE2 and its metabolites in the subjects with diabetic nephropathy.
